# Evaluation of mortality among marines and navy personnel exposed to contaminated drinking water at USMC base Camp Lejeune: a retrospective cohort study

**DOI:** 10.1186/1476-069X-13-10

**Published:** 2014-02-19

**Authors:** Frank J Bove, Perri Zeitz Ruckart, Morris Maslia, Theodore C Larson

**Affiliations:** 1 Division of Toxicology and Human Health Sciences, Agency for Toxic Substances and Disease Registry (ATSDR), 4770 Buford Highway, MS F-58, Atlanta, GA 30341, USA; 2 ATSDR, Division of Community Health Investigations, 4770 Buford Highway, MS F-59, Atlanta, GA 30341, USA

**Keywords:** Mortality, Cancers, Trichloroethylene, Tetrachloroethylene, Vinyl chloride, Benzene, Drinking water

## Abstract

**Background:**

Two drinking water systems at U.S. Marine Corps Base Camp Lejeune, North Carolina were contaminated with solvents during 1950s-1985.

**Methods:**

We conducted a retrospective cohort mortality study of Marine and Naval personnel who began service during 1975-1985 and were stationed at Camp Lejeune or Camp Pendleton, California during this period. Camp Pendleton’s drinking water was uncontaminated. Mortality follow-up was 1979-2008. Standardized Mortality Ratios were calculated using U.S. mortality rates as reference. We used survival analysis to compare mortality rates between Camp Lejeune (N = 154,932) and Camp Pendleton (N = 154,969) cohorts and assess effects of cumulative exposures to contaminants within the Camp Lejeune cohort. Models estimated monthly contaminant levels at residences. Confidence intervals (CIs) indicated precision of effect estimates.

**Results:**

There were 8,964 and 9,365 deaths respectively, in the Camp Lejeune and Camp Pendleton cohorts. Compared to Camp Pendleton, Camp Lejeune had elevated mortality hazard ratios (HRs) for all cancers (HR = 1.10, 95% CI: 1.00, 1.20), kidney cancer (HR = 1.35, 95% CI: 0.84, 2.16), liver cancer (HR = 1.42, 95% CI: 0.92, 2.20), esophageal cancer (HR = 1.43 95% CI: 0.85, 2.38), cervical cancer (HR = 1.33, 95% CI: 0.24, 7.32), Hodgkin lymphoma (HR = 1.47, 95% CI: 0.71, 3.06), and multiple myeloma (HR = 1.68, 95% CI: 0.76, 3.72). Within the Camp Lejeune cohort, monotonic categorical cumulative exposure trends were observed for kidney cancer and total contaminants (HR, high cumulative exposure = 1.54, 95% CI: 0.63, 3.75; log_10_ β = 0.06, 95% CI: -0.05, 0.17), Hodgkin lymphoma and trichloroethylene (HR, high cumulative exposure = 1.97, 95% CI: 0.55, 7.03; β = 0.00005, 95% CI: -0.00003, 0.00013) and benzene (HR, high cumulative exposure = 1.94, 95% CI: 0.54, 6.95; β = 0.00203, 95% CI: -0.00339, 0.00745). Amyotrophic Lateral Sclerosis (ALS) had HR = 2.21 (95% CI: 0.71, 6.86) at high cumulative vinyl chloride exposure but a non-monotonic exposure-response relationship (β = 0.0011, 95% CI: 0.0002, 0.0020).

**Conclusion:**

The study found elevated HRs at Camp Lejeune for several causes of death including cancers of the kidney, liver, esophagus, cervix, multiple myeloma, Hodgkin lymphoma and ALS. CIs were wide for most HRs. Because <6% of the cohort had died, long-term follow-up would be necessary to comprehensively assess effects of drinking water exposures at the base.

## Background

Samples taken during 1980-1985 at United States Marine Corps (USMC) Base Camp Lejeune, North Carolina detected solvents in drinking water supplied by two of the base’s eight treatment plants, Tarawa Terrace (TT) and Hadnot Point (HP). The TT supply wells were contaminated by an off-base dry cleaning business. The HP supply wells were contaminated by on-base sources: leaking underground storage tanks, industrial area spills and waste disposal sites. Contaminated supply wells in the TT and HP systems were shut down by February 1985 [1,2].

The primary contaminant in the TT distribution system was tetrachloroethylene (PCE) with a maximum measured level of 215 micrograms per liter (μg/L). Also detected were much lower levels of trichloroethylene (TCE), trans-1,2-dichloroethylene, and vinyl chloride, created when PCE degraded in ground water over time. The TT system served approximately 1,850 family housing units on base during 1975-1985 [1].

The primary contaminant in the HP distribution system was TCE with a maximum detected level of 1,400 μg/L. The maximum level of PCE was 100 μg/L, and benzene was also detected. Trans-1,2-dichloroethylene and vinyl chloride were present due to degradation of TCE in ground water [2]. During 1975-1985, the HP system served the “mainside” area of the base where a majority of bachelor’s quarters (“barracks”) and a few family housing units were located.

The Holcomb Boulevard system was a third system at the base, which served approximately 2,100 family housing units and was uncontaminated except for intermittent periods during dry spring-summer months when the HP system provided supplementary water. During a 2-week period in early 1985, the Holcomb Boulevard treatment plant shut down for repairs and the HP system provided water for its service area.

In each system, water from supply wells was mixed together at the treatment plant prior to distribution. Contamination levels in each system varied depending on the wells in use at a particular time.

Current U.S. maximum contaminant levels (MCLs) for TCE, PCE and benzene are 5 μg/L. The MCL for vinyl chloride is 2 μg/L. TCE has recently been classified as a human carcinogen [3,4]. Vinyl chloride and benzene are also classified as human carcinogens [5]. PCE is classified as a “likely” or “probable” human carcinogen [4,6].

Several meta-analyses and reviews assessed health effects of these chemicals [3-7]. Most of the evidence has come from occupational studies where the primary route of exposure was inhalation. Drinking water exposure to these chemicals involves contributions to total internal body dose from three routes: ingestion, inhalation and dermal. The dose from the inhalation and dermal routes may be as high as the dose from the ingestion route. For example, an internal dose via inhalation to TCE during a 10-minute shower may equal the internal dose via the ingestion of 2 liters of TCE-contaminated drinking water [8].

The literature is limited on health effects of drinking water exposures to these chemicals. A drinking water study in NJ observed associations between TCE and leukemia and non-Hodgkin lymphoma (NHL), and PCE and NHL [9]. PCE-contaminated drinking water was associated with lung cancer, bladder cancer, leukemia, rectal cancer, and female breast cancer in a study at Cape Cod, MA [10-12]. No studies have evaluated associations between drinking water exposures to these chemicals and medically confirmed, non-cancer diseases in adults.

The purpose of this study was to determine whether exposures of Marine and Naval personnel to contaminated drinking water at Camp Lejeune increased risk of mortality from cancers and other chronic diseases.

## Methods

We identified several diseases of *primary* interest: cancers of the kidney, hematopoietic system (NHL, leukemia, multiple myeloma, Hodgkin lymphoma), liver, bladder, esophagus and cervix. Kidney cancer, NHL and liver cancer were selected because the U.S. Environmental Protection Agency (EPA) and the International Agency For Research On Cancer cited evidence for a causal association with TCE exposure, although the evidence for liver cancer is “more limited” than the evidence for kidney cancer and NHL [4,7]. The National Toxicology Program (NTP) concluded that there was “evidence for consistent positive associations” between PCE and esophageal and cervical cancer, and EPA cited evidence for associations between PCE and bladder cancer and multiple myeloma [5,6]. Benzene is a known cause of leukemia.

Diseases of *secondary* interest were identified based on information from literature reviews suggesting possible associations with the contaminants or with solvents in general: aplastic anemia, amyotrophic lateral sclerosis (ALS), multiple sclerosis (MS), kidney and liver diseases, Parkinson’s disease, and cancers of the connective tissue, brain, pancreas, oral cavity, pharynx, lung, larynx, prostate, breast, colon and rectum [3,5-7,13].

Because this was a data linkage study with no smoking information, we evaluated smoking-related diseases not known to be associated with the contaminants to assess possible confounding: cardiovascular disease, chronic obstructive pulmonary disease (COPD), and stomach cancer.

### Study population and eligibility

The Camp Lejeune cohort consisted of 154,932 Marine and Naval personnel (“Marines”) who began active duty service during April 1975 – December 1985 and were stationed at Camp Lejeune anytime during this period. A comparison cohort consisted of 154,969 Marine and Naval personnel who began active duty service during April 1975 – December 1985, were stationed anytime during this period at USMC Base Camp Pendleton, but were not stationed at Camp Lejeune during this period. Camp Pendleton, located along the Southern California coast in northern San Diego County and southern Orange County, did not have contaminated drinking water during the period when the cohort was stationed at the base [14].

We obtained data for Camp Lejeune and Camp Pendleton from Defense Manpower Data Center (DMDC) Active Duty Military Personnel Master File for April 1975-December 1985. Unit information first became available in the DMDC file in April 1975 [15]. The USMC provided a list of units stationed at Camps Lejeune and Pendleton during 1975-1985. The quarterly DMDC file contained Social Security number (SSN), date of birth, sex, race/ethnicity, education, marital status, rank, active duty start date, total months of service, and military occupation code. This study was approved by the Centers for Disease Control and Prevention Institutional Review Board.

### Vital status ascertainment

Personal identifier information from the DMDC database was matched to data in the Social Security Administration (SSA) Death Master File (DMF) and SSA Office of Research, Evaluation and Statistics (ORES) Presumed Living Search to determine vital status [16,17]. For those not matched, a commercial tracing service was used to determine vital status. Identified deaths and individuals whose vital status remained unknown were then searched in the National Death Index (NDI). Those whose vital status remained unknown after the NDI search were considered “lost to follow-up” but contributed person-years to the study until the last date they were known to be alive based on commercial tracing or DMDC data. Underlying and contributing causes of death information were obtained from NDI.

### Exposure assessment

Due to limited numbers of historical samples for drinking water contamination, ATSDR conducted a historical reconstruction of the contamination using ground water fate and transport and distribution system models. Monthly average estimates of contaminant concentrations in each system were computed and reported in peer-reviewed agency reports [1,2]. Table 1 summarizes the estimated monthly mean contaminant concentrations from January 1975 through February 1985. Estimated monthly mean concentrations of PCE in the Tarawa Terrace distribution system during this period ranged from 0 to 158 μg/L with a median of approximately 85 μg/L (Table 1). PCE was the primary contaminant in the Tarawa Terrace system. Estimated monthly mean concentrations of TCE in the Hadnot Point distribution system during this period ranged from 0 to 783 μg/L, with a median level of approximately 366 μg/L (Table 2). TCE was the main contaminant in the Hadnot Point system although estimated monthly levels of PCE and vinyl chloride were often considerably above their MCLs, with medians of the estimates during this period of 15 μg/L and 22 μg/L, respectively.

**Table 1:** Estimated monthly average contaminant concentrations in the Tarawa Terrace system, 1975 – 1985

**1975 – 1985 (132 months)**					
**Contaminant**	**Mean (μg/L)**	**Median (μg/L)**	**Range (μg/L)**	**# Months >MCL**	**# Months >100 μg/L**
Tetrachloroethylene	75.7	84.9	0 – 158.1	117	16
Trichloroethylene	3.1	3.5	0 – 6.6	11	0
Vinyl chloride	5.6	6.2	0 – 12.3	117	0
**1975 – 1979 (60 months)**					
Tetrachloroethylene	68.3	68.2	43.8 – 94.8	60	0
Trichloroethylene	2.8	2.9	1.7 – 3.9	0	0
Vinyl chloride	5.2	5.5	2.6 – 7.3	60	0
January 1980 – January 1985 (61 months)*					
Tetrachloroethylene	96.1	95.5	0^¥^ – 158.1	57	16
Trichloroethylene	3.9	3.9	0^¥^ – 6.6	11	0
Vinyl chloride	7.0	7.0	0^¥^ – 12.3	57	0

*Two contaminated wells were shut down in January 1985. Estimated monthly average tetrachloroethylene levels from February through December 1985 were <4 μg/L.

^¥^One contaminated well was shut down for maintenance during 7/80 – 8/80 and 1/83 – 2/83. The other contaminated well was not in use until August 1984.

**Table 2:** Estimated monthly average contaminant concentrations in the Hadnot Point system, 1975 – 1985

**1975 – 1985**					
**Contaminant**	**Mean (μg/L)**	**Median (μg/L)**	**Range (μg/L)**	**# Months >MCL**	**# Months >100 μg/L**
Tetrachloroethylene	15.7	15.4	0 – 38.7	111	0
Trichloroethylene	358.7	365.9	0 – 783.3	122	113
Vinyl chloride	24.0	22.2	0 – 67.3	122	0
Benzene	5.4	4.6	0 – 12.2	63	0
**1975 – 1979**					
Tetrachloroethylene	12.2	12.0	1.4 – 24.1	53	0
Trichloroethylene	325.1	327.7	60.6 – 546.3	60	55
Vinyl chloride	17.3	16.5	2.3 – 33.4	60	0
Benzene	3.5	3.4	0 – 5.8	4	0
January 1980 – February 1985*					
Tetrachloroethylene	21.5	21.4	2.2 – 38.7	58	0
Trichloroethylene	449.2	446.2	42.6 – 783.3	62	58
Vinyl chloride	34.3	35.7	4.2 – 67.3	62	0
Benzene	7.6	7.6	1.6 – 12.2	59	0

*Contaminated wells were shut down after February 1985. From March through December 1985, estimated monthly average levels of trichloroethylene, tetrachloroethylene and vinyl chloride were <1 μg/L, and benzene was <4 μg/L.

**Figure 1:**
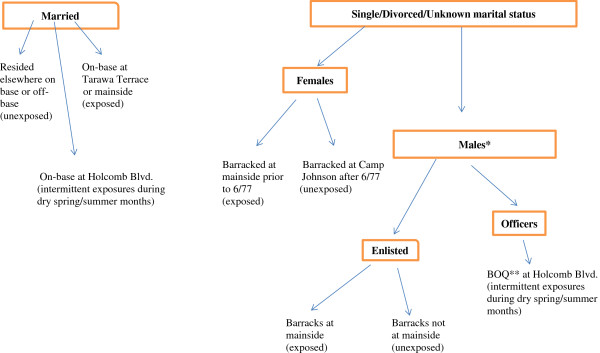
**Camp Lejeune cohort exposure assessment.** *8th Marines (both enlisted and officers) moved to Camp Geiger (an unexposed area) by 1980. **BOQ = Bachelor officer quarters (Note: BOQs elsewhere on base were in unexposed areas).

On average, an individual in the Camp Lejeune cohort resided at the base for 18 months. Each individual was assigned estimated monthly average contaminant concentrations in the drinking water system serving the individual’s residence during the period of residence. We used several sources of information to determine an individual’s residence (Figure 1).

Married Camp Lejeune cohort members resided either in base family housing or in off-base housing. We used probability and manual matching to link married cohort members to base family housing records on name, rank, occupancy dates, and dates stationed on base.

Unmarried officers resided in bachelor officers’ quarters served by the Holcomb Boulevard water system during 1975-1985. Unmarried enlisted individuals resided in barracks. Unit barrack locations were identified using information provided by retired marines, base staff, and base command chronologies. Female marines resided in areas served by the HP system until June 1977 when they moved to an area with uncontaminated drinking water.

### Data analysis

Follow-up began on January 1, 1979 or start of active duty service at either base, whichever was later, and continued until December 31, 2008, if the person was known to be alive, or to date of death. Those with unknown vital status were followed until the last date they were known to be alive based on available data.

We used the Life Table Analysis System (LTAS) to compute cause-specific, standardized mortality ratios (SMRs) and 95% confidence intervals (CIs) comparing the Camp Lejeune and Camp Pendleton cohorts to age- sex- race-and calendar period-specific U.S. mortality rates for underlying and contributing causes of death [18]. In apportioning person-years to specific age-race-sex-and calendar period categories for each base, once an individual was stationed at Camp Lejeune (e.g., some began at Camp Pendleton and later transferred to Camp Lejeune), all subsequent person-years were assigned to Camp Lejeune.

a. Comparisons between Camp Lejeune and Camp Pendleton cohorts

We used Cox extended regression models [19] with age as the time variable and base location as a time-varying dichotomous variable to calculate hazard ratios (HRs) comparing mortality rates between Camp Lejeune and Camp Pendleton cohorts. These analyses assumed everyone at Camp Lejeune was exposed to contaminated drinking water at their residences and/or during daily activities on base while those at Camp Pendleton were unexposed.

We accounted for a “latency period” by lagging exposure to a base by 10, 15, and 20 years in addition to an analysis with no lag. For example, a 10 year lag would assign to an individual aged 29, the base the individual was stationed at age 19. If this individual was not yet serving at age 19, then the person-year for age 29 was assigned to a category, “not at either base”. We used the Akaike’s information criterion (AIC), a measure of model goodness of fit, to select an appropriate lag period.

b. Analyses within the Camp Lejeune cohort

Within the Camp Lejeune cohort, we evaluated exposure-response relationships between cumulative exposures to drinking water contaminants and cause of mortality using Cox extended regression models with age as the time variable and cumulative exposure as a time-varying variable. Estimated monthly average contaminant concentrations in the water system serving the individual’s residence and occupancy dates were used to calculate cumulative exposures (“μg/L-months”) to each contaminant and to the total amount of these contaminants (“TVOC”).

We evaluated untransformed and log_10_ transformed cumulative exposures as continuous variables. The log transform is appropriate when exposure-response relationships plateau or attenuate at higher levels of exposure [20]. We added a small constant (i.e., 0.001) to the monthly average contaminant concentrations to avoid taking the logarithm of zero. A one unit increase in the log-transformed cumulative exposure corresponds to a ten-fold increase in cumulative exposure.

We also evaluated cumulative exposure as categorical variable (no, low, medium, and high exposure) based on cumulative exposure distributions of each contaminant among those exposed cohort members who died of any cancer. The low to high exposure categories contained approximately equal numbers of exposed cancer deaths in order to produce similar variances for hazard ratios across exposure categories [20].

We evaluated PCE, TCE, vinyl chloride, and benzene separately because the contaminants were highly correlated and could not be included together in a model. For example, correlations ≥ .96 were observed between cumulative exposures to TCE, VC, and benzene because the Hadnot Point system was the source of higher levels of these contaminants. Lower correlations ranging from .44 to .53 were observed between PCE and the other contaminants because the Tarawa Terrace system had high levels of PCE but low levels of other contaminants. Because of the high correlations among the contaminants, it is not possible to separate the effects of each of the individual contaminants, although TCE and PCE levels were substantially higher than the levels of the other contaminants. In order to evaluate the contaminants as a group, we created the variable, TVOC, by combining PCE, TCE, trans-1,2-dichloroethylene, vinyl chloride and benzene.

To account for latency, we evaluated 10, 15, and 20 year lag periods for cumulative exposures in addition to a “no lag” period.

The use of either categorical or continuous exposure variables (whether transformed or not) imposes a structure on the exposure-response relationship which may be inaccurate [20]. To obtain a more flexible, smoothed exposure-response curve, we specified a restricted cubic spline (RCS) function for cumulative exposure in the Cox extended model [21]. Four knots were located at the 5^th^, 25^th^, 75^th^, and 95^th^ percentiles among those with cumulative exposure to a contaminant >1 μg/L-months. The RCS function allowed the shape of the HR curve to vary within and between these knots and restricted the curve to be linear before the first knot and after the last knot. The resulting curve is useful for assessing whether the exposure-response relationship is adequately captured by either the categorical or continuous exposure variables.

In subsequent analyses, we evaluated duration at Camp Lejeune and duration exposed to the contaminated drinking water as time-varying categorical variables, and average exposures as time-independent categorical and continuous variables.

c. Confounder assessment

DMDC and NDI data were available for sex, race, marital status, birth cohort, date of death, age at death, rank, education, and duty occupation. For confounding to occur, a risk factor must be associated with the exposure as well as with the disease of interest. To identify potential confounding, we used a “10% change in the estimate” rule [22]. Final Cox extended models included sex, race, rank, and education.

Information on smoking, alcohol consumption, and occupational history prior to or after active duty service, was unavailable. We evaluated possible smoking confounding by subtracting the log HR among smoking-related diseases from the log HR of the disease of interest [23].

Because the cohorts began active duty service after 1974, none were Vietnam veterans. However, information was unavailable concerning service in later wars involving hazardous exposures.

d. Interpretation of findings

Interpretation of study findings was based on the magnitude of the adjusted SMR or HR. For analyses internal to the Camp Lejeune cohort, we also based our interpretation on the exposure-response relationship, giving more emphasis to monotonic trends in the categorical cumulative exposure variables. A monotonic trend occurs when every change in the HR with increasing category of exposure is in the same direction, although the trend could have flat segments but never reverse direction [24]. Because exposure-response trends could be distorted by biases such as exposure misclassification, we also emphasized non-monotonic exposure-response trends when an elevated HR was observed in the high exposure group.

We computed 95% confidence intervals to show the precision of the HR and regression coefficient estimates, and we included p-values for information purposes only. We did not use statistical significance testing to interpret findings [24-28].

## Results and discussion

The cohorts had similar demographics and most were under age 55 by the end of follow-up (Table 3). Each cohort contributed approximately 4 million person-years of follow-up, about 6% died during the follow-up period, and vital status was unknown for less than 2%.

**Table 3:** Demographics of the Camp Lejeune and Camp Pendleton cohorts

**Factor**	**Camp Lejeune**	**Camp Pendleton**
	**N = 154,932**	**N = 154,969**
Male	94.8%	96.4%
Female	5.2%	3.6%
“White”	73.1%	77.6%
African American	24.2%	17.0%
“other” or unknown	2.7%	5.4%
Median age, start of follow-up	20	20
Median age, end of follow-up	49	49
% ≥55 yrs, end of follow-up	2.7%	3.2%
Not a high school graduate	11.3%	14.7%
High school graduate	84.9%	80.5%
College graduate	3.8%	4.8%
Enlisted	96.4%	95.5%
Officer	3.6%	4.5%
Median months active duty service	36	35
Total deaths	8,964 (5.8%)	9,365 (6.0%)
% deaths occurring >1995	55.5%	54.7%
Total lost to follow-up	1,990 (1.3%)	2,339 (1.5%)
Total person-years of follow-up	4.14 million	4.19 million

### Standardized mortality ratio (SMR) analyses

Because we observed similar results for contributing and underlying causes of death, only results for underlying cause of death are presented. Over a quarter of deaths in both cohorts were due to cancers and cardiovascular diseases combined (Table 4). Suicide, homicide, transportation injuries and other injuries accounted for about half of deaths in the cohorts (data not shown).

**Table 4:** Standardized mortality ratios (SMRs), underlying cause of death

**Underlying**	**Camp Pendleton (reference)**			**Camp Lejeune**		
Cause of death	Obs.	Exp.	SMR (95% CI)	Obs.	Exp.	SMR (95% CI)
All causes	9,365	10,922	0.86 (0.84, 0.87)	8,964	10,864	0.83 (0.81, 0.84)
All cancers	1,008	1,296	0.78 (0.73, 0.83)	1,078	1,272	0.85 (0.80, 0.90)
Diseases of primary interest						
Kidney cancer	33	37.20	0.89 (0.61, 1.25)	42	36.08	1.16 (0.84, 1.57)
Bladder cancer	14	13.65	1.03 (0.56, 1.72)	11	13.04	0.84 (0.42, 1.51)
Liver* cancer	39	69.21	0.56 (0.40, 0.77)	51	69.20	0.74 (0.55, 0.97)
Esophageal cancer	27	43.33	0.62 (0.41, 0.91)	35	41.34	0.85 (0.59, 1.18)
Hematopoietic cancers	167	215.93	0.77 (0.66, 0.90)	165	211.10	0.78 (0.67, 0.91)
Hodgkin	23	25.86	0.89 (0.56, 1.33)	24	25.03	0.96 (0.61, 1.43)
NHL**	68	87.56	0.78 (0.60, 0.98)	58	85.50	0.68 (0.52, 0.88)
Multiple myeloma	12	16.26	0.74 (0.38, 1.29)	17	16.13	1.05 (0.61, 1.69)
Leukemias	64	86.26	0.74 (0.57, 0.95)	66	84.43	0.78 (0.60, 0.99)
Cervical cancer	2	3.53	0.57 (0.07, 2.05)	5	4.88	1.03 (0.33, 2.39)
**Diseases of secondary interest**						
Pancreatic cancer	44	60.05	0.73 (0.53, 0.98)	57	58.29	0.98 (0.74, 1.27)
Colon cancer	73	93.28	0.78 (0.61, 0.98)	86	92.29	0.93 (0.75, 1.15)
Rectal cancer	16	29.84	0.54 (0.31, 0.87)	24	29.54	0.81 (0.52, 1.21)
Soft tissue cancers	21	27.82	0.75 (0.47, 1.15)	29	27.44	1.06 (0.71, 1.52)
Brain cancer	80	93.36	0.86 (0.68, 1.07)	74	88.95	0.83 (0.65, 1.04)
Laryngeal cancer	13	12.15	1.07 (0.57, 1.83)	6	11.92	0.50 (0.18, 1.10)
Lung*** cancer	216	265.44	0.81 (0.71, 0.93)	237	259.01	0.92 (0.80, 1.04)
Oral cancers****	35	37.64	0.93 (0.65, 1.29)	26	37.38	0.70 (0.45, 1.02)
Breast (female) cancer	7	14.68	0.48 (0.19, 0.98)	10	19.62	0.51 (0.24, 0.94)
Prostate cancer	15	10.68	1.41 (0.79, 2.32)	18	10.41	1.73 (1.02, 2.73)
Liver diseases	233	322.70	0.72 (0.63, 0.82)	191	311.90	0.61 (0.53, 0.71)
Kidney diseases	37	71.72	0.52 (0.37, 0.71)	37	74.54	0.50 (0.35, 0.68)
ALS	27	19.42	1.39 (0.92, 2.02)	21	18.45	1.14 (0.70, 1.74)
Multiple sclerosis	10	14.95	0.67 (0.32, 1.23)	12	14.75	0.81 (0.42, 1.42)
**Smoking-related diseases (not known to be related to solvent exposure)**						
Stomach cancer	29	41.43	0.70 (0.47, 1.01)	35	41.88	0.84 (0.58, 1.16)
Cardiovascular disease†	1,376	1,791	0.77 (0.73, 0.81)	1,390	1,781	0.78 (0.74, 0.82)
COPD	45	55.82	0.81 (0.59, 1.08)	47	53.89	0.87 (0.64, 1.16)

Not evaluated due to small numbers were Parkinson’s disease and male breast cancer.

*Biliary passages, liver and gall bladder **Non-Hodgkin lymphoma.

***Trachea, bronchus, and lung ****Oral cavity and Pharynx.

†Includes diseases of the heart and other diseases of the circulatory system.

Camp Lejeune = 154,932; person-years = 4,140,042.

Camp Pendleton = 154,969; person-years = 4,190,132.

Comparing each cohort to U.S. mortality rates, most SMRs were less than 1.00 indicating a “healthy veteran effect” [29] for cancers and non-cancers (Table 4). For diseases of primary interest, we observed SMRs above 1.00 in the Camp Lejeune cohort for kidney cancer (SMR = 1.16, 95% CI: 0.84, 1.57), multiple myeloma (SMR = 1.05, 95% CI: 0.61, 1.69), and cervical cancer (SMR = 1.03, 95% CI: 0.33, 2.39). At Camp Pendleton, the only disease of primary interest with an SMR greater than 1.00 was bladder cancer (SMR = 1.03, 95% CI: 0.56, 1.72). For diseases of secondary interest, both Camp Lejeune and Camp Pendleton cohorts had SMRs > 1.00 for prostate cancer (SMR = 1.73, 95% CI: 1.02, 2.73); and SMR = 1.41, 95% CI: 0.79, 2.32, respectively) and ALS (SMR = 1.14, 95% CI: 0.70, 1.74; and SMR = 1.39, 95% CI: 0.92, 2.02, respectively). Soft tissue sarcoma was elevated in the Camp Lejeune cohort (SMR = 1.06, 95% CI: 0.71, 1.52) and cancer of the larynx was elevated in the Camp Pendleton cohort (SMR = 1.07, 95% CI: 0.57, 1.83). SMRs for male breast cancer and Parkinson’s disease were not calculated because there were <5 cases in each cohort. We did not calculate SMRs for aplastic anemia because LTAS combined aplastic anemia with other anemias.

### Comparison of Camp Lejeune with Camp Pendleton

Table 5 presents results for comparisons of mortality between the two cohorts. A 10 year lag generally had the lowest AIC values. Camp Lejeune had an elevated HR for “all cancers” (HR = 1.10, 95% CI: 1.00, 1.20). For diseases of primary interest, Camp Lejeune had elevated HRs for kidney cancer (HR = 1.35, 95% CI: 0.84, 2.16), liver cancer (HR = 1.42, 95% CI: 0.92, 2.20), esophageal cancer (HR = 1.43, 95% CI: 0.85, 2.38), multiple myeloma (HR = 1.68, 95% CI: 0.76, 3.72), leukemias (HR = 1.11, 95% CI: 0.75, 1.62), Hodgkin lymphoma (HR = 1.47, 95% CI: 0.71, 3.06), and cervical cancer (HR = 1.33, 95% CI: 0.24, 7.32). Bladder cancer and NHL had HRs < 1.00.

**Table 5:** Camp Lejeune vs Camp Pendleton: hazard ratios and 95% confidence intervals, adjusted by sex, race, rank and education, 10-year lag

**Underlying cause of death**	**Hazard ratio**	**95% LCL**	**95% UCL**	**p-value**
All cancers	1.10	*1.00*	*1.20*	0.02
**Diseases of primary interest**				
Kidney cancer	1.35	*0.84*	*2.16*	0.19
Bladder cancer	0.76	*0.34*	*1.71*	0.50
Liver* cancer	1.42	*0.92*	*2.20*	0.11
Esophageal cancer	1.43	*0.85*	*2.38*	0.17
Hematopoietic cancers	1.05	*0.82*	*1.33*	0.57
Hodgkin	1.47	*0.71*	*3.06*	0.26
NHL**	0.81	*0.56*	*1.18*	0.43
Multiple myeloma	1.68	*0.76*	*3.72*	0.21
Leukemias	1.11	*0.75*	*1.62*	0.63
Cervical cancer	1.33	*0.24*	*7.32*	0.74
**Diseases of secondary interest**				
Pancreatic cancer	1.36	*0.91*	*2.02*	0.13
Colorectal cancers	1.13	*0.85*	*1.51*	0.35
Colon cancer	1.04	*0.75*	*1.43*	0.76
Rectal cancer	1.60	*0.83*	*3.07*	0.15
Soft tissue cancers	1.38	*0.73*	*2.64*	0.30
Brain cancer	0.93	*0.67*	*1.30*	0.84
Laryngeal cancer	0.54	*0.20*	*1.45*	0.22
Lung*** cancer	1.16	*0.96*	*1.40*	0.10
Oral cancers****	0.82	*0.48*	*1.41*	0.46
Breast (female) cancer	0.93	*0.34*	*2.50*	0.88
Prostate cancer	1.23	*0.60*	*2.49*	0.57
Liver diseases	0.87	*0.71*	*1.06*	0.18
Kidney diseases	1.00	*0.63*	*1.63*	0.95
ALS	0.83	*0.47*	*1.48*	0.54
Multiple sclerosis	1.21	*0.50*	*2.94*	0.65
**Smoking-related diseases (not known to be related to solvent exposure)**				
Stomach cancer	1.15	*0.70*	*1.90*	0.58
Cardiovascular disease†	1.04	*0.95*	*1.11*	0.31
COPD	1.08	*0.70*	*1.67*	0.70

Not evaluated due to small numbers were Parkinson’s disease, male breast cancer, and aplastic anemia.

LCL: lower confidence limit UCL: upper confidence limit.

*Biliary passages, liver and gall bladder ***Trachea, bronchus, and lung.

****Oral cavity and Pharynx.

†Includes heart diseases and other diseases of the circulatory system.

An evaluation of leukemia subtypes was not conducted because a considerable percentage (22.7%) of the leukemias were classified as “acute leukemia, not otherwise specified” in the Camp Lejeune cohort compared to the percentage (9.4%) occurring in the Camp Pendleton cohort.

We conducted additional analyses to determine whether the elevated HRs for the Camp Lejeune cohort could be explained by cumulative exposures to the contaminants or by some other factor. For these analyses, the Camp Pendleton cohort was the reference group and the Camp Lejeune cohort was split into two groupings: no/low cumulative exposure and medium/high cumulative exposure (Additional file 1: Table S3). For example, if HRs in the no/very low cumulative exposure group were higher than HRs in the medium/high cumulative exposure group, then the elevation could be due to some other factor. For kidney cancer, Hodgkin lymphoma and leukemias, those with no/very low cumulative exposures had HRs ≤ 1.00 with all of the elevation in risk occurring among those with higher cumulative exposures. For cervical cancer, the HRs were ≤1.12 among those with no/very low cumulative exposures, while the HRs were >5.80 among those with higher cumulative exposures. For multiple myeloma, elevated HRs did occur among those with no/very low cumulative exposures, ranging from 1.10 to 1.40, while HRs ranging from 1.60 to 1.70 occurred among those with higher cumulative exposures. For liver cancer, the HRs for no/very low and higher cumulative exposures were similar, ranging from 1.30 to 1.40, while for esophageal cancer, the no/very low cumulative exposure group had much higher HRs than the higher exposure group.

Of diseases of secondary interest, Camp Lejeune had elevated HRs for colorectal cancers, in particular, rectal cancer (HR = 1.60, 95% CI: 0.83, 3.07), pancreatic cancer (HR = 1.36, 95% CI: 0.91, 2.02), soft tissue cancers (HR = 1.38, 95% CI: 0.73, 2.64), lung cancer (HR = 1.16, 95% CI: 0.96, 1.40), prostate cancer (HR = 1.23, 95% CI: 0.60, 2.49), and multiple sclerosis (HR = 1.21, 95% CI: 0.50, 2.94). Diseases with HRs ≤ 1.00 were ALS, liver diseases, kidney diseases and brain, laryngeal and oral cancers.

The elevation in the HR for lung cancer was due entirely to those with higher cumulative exposures at Camp Lejeune (Additional file 1: Table S3). For rectal cancer, the HRs were similar for the no/very low and higher cumulative exposure groups. For soft tissue cancers, pancreatic cancer, prostate cancers and multiple sclerosis, the elevation in HRs was due primarily to those with very low cumulative exposures.

The highest HR among smoking-related diseases was for stomach cancer (HR = 1.15, 95% CI: 0.70, 1.90). Using the stomach cancer result to adjust for smoking confounding would reduce the HRs for diseases of primary and secondary interest by 13%. However, HRs for the other smoking-related diseases (COPD and cardiovascular disease) were less than 1.10, and HRs for diseases that are both smoking and solvent related (e.g., laryngeal and oral cancers) were less than 1.00. Therefore it is likely that the confounding effects of smoking are less than 10% for the comparisons between Camp Lejeune and Camp Pendleton.

### Analyses internal to the Camp Lejeune cohort

Categorizations of cumulative exposure (“μg/L –months”) for each contaminant are presented in Table 6. Full results for categorical and continuous cumulative exposures are in the Additional file 2: Table S1, Additional file 3: Table S2. Similar AIC values were observed for the exposure lag and no lag periods evaluated so a 10 year lag was selected. The reference group consisted of Camp Lejeune cohort members with cumulative exposures within the reference levels listed in Table 6. Both the reference group and the low cumulative exposure category had a higher percentage of females, “white” race, officers, and college graduates than the medium and high cumulative exposure categories. All analyses included these variables in the models.

**Table 6:** Categorization of cumulative exposure variables (μg/L –months) within the Camp Lejeune cohort

**Category**	**Reference level**	**Low exposure**	**Medium exposure**	**High exposure**
**Cumulative tetrachloroethylene (for >1 μg/L –months: mean = 402.6, median = 269.5)**				
Level*	≤ 1	>1 - 155	>155 - 380	>380 – 8,585
**Number (%)**	66, 582 (43.0%)	28,230 (18.2%)	27,255 (17.6%)	32,865 (21.2%)
**Cumulative trichloroethylene (for >1 μg/L –months: mean = 6,369.3, median = 5,289.0)**				
Level*	≤ 1	>1 – 3,100	>3,100 – 7,700	>7,700 – 39,745
**Number (%)**	64, 584 (41.7%)	31,069 (20.1%)	27,638 (17.8%)	31,641 (20.4%)
**Cumulative vinyl chloride (for >1 μg/L –months: mean = 458.9, median = 360.6)**				
Level*	≤ 1	>1 - 205	>205 - 500	>500 – 2,800
**Number (%)**	66, 470 (42.9%)	27,651 (17.8%)	28,063 (18.1%)	32,748 (21.1%)
**Cumulative benzene (for ≥2 μg/L –months: mean = 104.7, median = 83.2)**				
Level*	< 2	2 - 45	>45 - 110	>110 - 601
**Number (%)**	64, 580 (41.7%)	24,579 (15.9%)	31,838 (20.5%)	33,935 (21.9%)
**Cumulative TVOC (for >1 μg/L –months: mean = 9,605.1, median = 7,652.8)**				
Level*	≤ 1	>1 – 4,600	>4,600 – 12,250	>12,250 - 64,016
**Number (%)**	57, 328 (37.0%)	35,432 (22.9%)	29,687 (19.2%)	32,485 (21.0%)

*An individual’s maximum amount of cumulative exposure (μg/L –months**)** at the end of follow-up.

N = 154,932.

We observed a monotonic exposure-response relationship for kidney cancer and the categorized cumulative exposure variable for TVOC (HR for high exposure category = 1.54, 95% CI: 0.63, 3.75) (Table 7a). A non-monotonic exposure-response trend was observed for PCE and Kidney cancer (HR for high exposure category = 1.59, 95% CI: 0.66, 3.86). Non-monotonic and weaker effects were seen for other contaminants. The log_10_ transform had lower AIC values indicating attenuation of HRs at higher exposure levels [20], and this attenuation was reflected in the spline for TVOC and kidney cancer with HRs rising in a linear fashion to a peak value of 1.7 in mid-range level of cumulative exposure before declining at higher exposure levels. (see Additional file 4: Figure S1). The regression coefficients for the log_10_ transform of cumulative exposure to PCE and TVOC were 0.0813 (95% CI: -0.0553, 0.2179) and 0.0633 (95% CI: -0.0481, 0.1747), respectively.

**Table 7:** Hazard ratios (95% CI) for categorical cumulative exposure, and coefficients (95% CI) for continuous cumulative exposure

	**Low exposure**	**Medium exposure**	**High exposure**	**Cumulative exposure**	**Log**_**10 **_**cumulative exposure**
**a. Kidney cancer (N=42)**					
PCE	1.40 (0.54, 3.58) N=8	1.82 (0.75, 4.42) N=11	1.59 (0.66, 3.86) N=11	.00009 (-0.00048, 0.00065), p=.76	.0813 (-0.0553, 0.2179), p=.24
TVOC	1.42 (0.58, 3.47) N=10	1.44 (0.58, 3.59) N=10	1.54 (0.63, 3.75) N=11	.00001 (-0.00003, 0.00005) p=.59	.0633 (-0.0481, 0.1747) p=.26
**b. Hodgkin lymphoma (N=24)**					
TCE	1.52 (0.42, 5.59) N=4	1.63 (0.43, 6.12) N=4	1.97 (0.55, 7.03) N=5	.00005 (-0.00003, 0.00013) p=.20	.0940 (-0.0650, 0.2530) p=.25
VC	1.20 (0.29, 4.94) N=3	2.07 (0.59, 7.27) N=5	1.99 (0.56, 7.13) N=5	.00056 (-0.00060, 0.00172) p=.34	.1101 (-0.0817, 0.3019) p=.26
Benzene	1.24 (0.30, 5.11) N=3	1.88 (0.54, 6.61) N=5	1.94 (0.54, 6.95) N=5	.00203 (-0.00339, 0.00745) p=.46	.1074 (-0.1088, 0.3236) p=.33
TVOC	0.66 (0.13, 3.39) N=2	1.77 (0.50, 6.25) N=5	2.17 (0.63, 7.50) N=6	.00003 (-0.00003, 0.00009) p=.24	.0752 (-0.0818, 0.2322) p=.35
**c. Leukemias (N=66)**					
TCE	2.00 (1.00, 4.00) N=16	1.54 (0.71, 3.36) N=11	1.81 (0.85, 3.85) N=13	.00002 (-0.00004, 0.00008) p=.46	.0801 (-0.0093, 0.1695) p=.08
Benzene	2.54 (1.27, 5.08) N=17	1.46 (0.66, 3.20) N=11	1.69 (0.77, 3.67) N=12	.00168 (-0.00158, 0.00494) p=.31	.1276 (0.0020, 0.2532) p=.05
TVOC	2.50 (1.24, 5.03) N=19	1.33 (0.56, 3.14) N=9	2.33 (1.08, 5.03) N=15	.00001 (-0.00003, 0.00005) p=.44	.0950 (0.0032, 0.1868) p=.04
**d. ALS (N=21)**					
TCE	0.91 (0.25, 3.23) N=4	0.87 (0.21, 3.57) N=3	1.93 (0.65, 5.79) N=8	.00007 (0.00001, 0.00013) p=.04	.0436 (-0.1083, 0.1955) p=.57
PCE	0.69 (0.13, 3.55) N=2	1.58 (0.45, 5.50) N=5	1.96 (0.64, 6.02) N=8	.00039 (-0.00002, 0.00080) p=.06	.0836 (-0.1060, 0.2732) p=.39
VC	1.22 (0.33, 4.51) N=4	0.91 (0.22, 3.87) N=3	2.21 (0.71, 6.86) N=8	.00110 (0.00020, 0.00200) p=.02	.0724 (-0.1149, 0.2597) p=.45
TVOC	1.27 (0.37, 4.41) N=5	0.89 (0.21, 3.82) N=3	2.11 (0.67, 6.68) N=8	.00005 (0.00001, 0.00009) p=.03	.0702 (-0.0872, 0.2276) p=.38

**TCE**: Trichloroethylene **PCE**: Tetrachloroethylene (or perchloroethylene) **VC**: Vinyl chloride.

**TVOC**: (“total volatile organic compounds”) the sum of all contaminants (TCE, PCE, trans-1,2-dichloroethylene, vinyl chloride and benzene) in the drinking water.

The referent group for each contaminant consists of those Camp Lejeune cohort members with cumulative exposures within the reference level for that contaminant shown in Table 6.

Exposure lagged 10 years. Adjusted for race, sex, rank and education. Selected causes of death. Camp Lejeune cohort (N = 154,932).

We observed monotonic exposure-response relationships for Hodgkin lymphoma and TCE and benzene with HRs at the high exposure category of 1.97 (95% CI: 0.55, 7.03) and 1.94 (95% CI: 0.54, 6.95), respectively (Table 7b). A non-monotonic relationship was found for vinyl chloride and TVOC with HRs at the high exposure category of 1.99 (95% CI = 0.56, 7.13) and 2.17 (95% CI: 0.63, 7.50), respectively. Similar AIC values were observed for untransformed and log_10_ transformed cumulative exposures. The regression coefficients for cumulative exposures to TCE and benzene were 0.00005 (95% CI: -0.00003, 0.00013) and 0.00203 (95% CI: -0.00339, 0.00745), respectively. The spline for TCE supported the categorized results as the HRs steadily increased in a linear fashion to approximately 2.4 in the high cumulative exposure range and then fell slightly thereafter (see Additional file 4: Figure S2).

Non-monotonic exposure-response relationships were observed for leukemias, with HRs for the high exposure category of 2.33 (95% CI: 1.08, 5.03), 1.81 (95% CI: 0.85, 3.85) and 1.69 (95% CI: 0.77, 3.67) for TVOC, TCE, and benzene, respectively (Table 7c). Lower AIC values were observed for log_10_ transformed cumulative exposures to TCE, benzene and TVOC with regression coefficients of 0.080 (95% CI: -0.009, 0.170), 0.128 (95% CI: 0.002, 0.253), and 0.095 (95% CI: 0.003, 0.187), respectively.

Two other diseases of primary interest had HRs above 1.00 in the high exposure category but trends were non-monotonic: NHL had HRs between 1.10 and 1.20 for TVOC, TCE, vinyl chloride and PCE, and bladder cancer had an HR of 2.26 for benzene based on 3 cases, and HRs of 1.20 for TVOC and PCE (see Additional file 2: Table S1). Multiple myeloma, liver cancer and esophageal cancer had HRs ≤1.00 in the high exposure category for each contaminant. Cervical cancer could not be evaluated because there were only 5 deaths in the Camp Lejeune cohort.

Of diseases of secondary interest, ALS had HRs > 1.90 in the high cumulative exposure category for TVOC (HR = 2.11, 95% CI: 0.67, 6.68), TCE (HR = 1.93, 95% CI: 0.65, 5.79), PCE (HR = 1.96, 95% CI: 0.64, 6.02), and vinyl chloride (HR = 2.21, 95% CI: 0.71, 6.86) but the exposure-response trends were not monotonic (Table 7d). The splines for these contaminants and ALS had similar exposure-response trends as those observed for the categorized cumulative exposure variables. For example, the spline for cumulative exposure to vinyl chloride indicated HRs < 1.00 until the high exposure range and then rose in a linear fashion to HRs >3.00 (Additional file 4: Figure S3a). Splines for TCE and TVOC were similar to the spline for vinyl chloride. For PCE, HRs >1.00 were observed near the end of the middle exposure range and rose linearly to approximately 3.50, leveling off thereafter (Additional file 4: Figure S3b). For all the contaminants, the lower AIC values were observed for untransformed cumulative exposures with regression coefficients for TCE, PCE, vinyl chloride and TVOC of 0.00007 (95% CI: 0.00001, 0.00013), 0.00039 (95% CI: -0.00002, 0.00080), 0.00110 (95% CI: 0.00020, 0.00200), and 0.00005 (95% CI: 0.00001, 0.00009), respectively.

We did not observe monotonic exposure-response trends for other diseases of secondary interest. The HR for PCE in the high exposure category and oral cancers was 1.80 (95% CI: 0.59, 5.46), but this was slightly lower than the HR at the low exposure category (HR = 1.89, 95% CI: 0.63, 5.66) and the middle exposure category had an HR < 1.00 (see Additional file 2: Table S1). Other diseases of secondary interest had HRs ≤1.20 (see Additional file 2: Table S1). Laryngeal cancer had too few cases (N = 4) to evaluate.

Except for benzene, we observed monotonic exposure-response relationships for the categorized cumulative exposure variables and cardiovascular disease, with HRs ≤ 1.12 in the high exposure categories. For stomach cancer and PCE, a non-monotonic relationship was observed with HR = 1.56 (95% CI: 0.66, 3.69) at the high exposure category. The HRs for COPD were <1.00 for the middle and high cumulative exposure categories of the contaminants (see Additional file 2: Table S1).

Analyses of duration of exposure and average exposure produced results similar to cumulative exposure and are not presented.

## Discussion

The diseases of primary and secondary interest under evaluation were selected based primarily on evidence from occupational studies of solvents such as TCE. Although occupational exposures occur primarily via inhalation and levels are generally much higher than drinking water exposures, the levels of TCE in the Hadnot Point distribution system were sufficiently high to result in exposures comparable to those that may occur in some occupational settings.

For example, daily inhalation exposures to TCE between 2.2 mg/day and 9.5 mg/day could occur in occupational settings where personal monitoring measurements indicated TCE air concentrations between 1.2 and 5.1 parts per million (ppm) [3]. A marine in training under warm weather conditions could drink between 1 and 2 quarts of water per hour [30]. Combining this ingestion rate with dermal and inhalation exposures from showering twice a day, a marine could consume a liter-equivalent of up to 8 liters of drinking water per day [31]. The Hadnot Point distribution system had a median TCE monthly average level of 446 μg/L during January 1980- February 1985 (see Table 2), thus resulting in a possible daily exposure as high as 3.6 mg/day, i.e., within the range of workday exposures that occurred in some occupational settings.

One estimate of mean TCE air concentrations across all industries from the 1950s through the 1980s was 38 parts per million (ppm) [32]. This level of exposure would be considerably higher than an exposure to a marine consuming Hadnot Point drinking water. However, TCE concentrations in industry have decreased over time in the U.S. By the 1980s, the geometric mean concentration of TCE in Danish industries was approximately 4.3 ppm [3,33], and this level of air concentration of TCE would result in exposure comparable to the drinking water exposure to TCE at Camp Lejeune. A meta-analysis of occupational studies conducted by EPA that evaluated “any TCE exposure” obtained RRs of 1.27, 1.23 and 1.29 for kidney cancer, NHL, and liver cancer, respectively [7]. Similar findings were observed in this study for kidney cancer and liver cancer, but not for NHL, when the Camp Lejeune cohort was compared to the Camp Pendleton cohort.

In the comparison between Camp Lejeune and Camp Pendleton, the HRs for several cancers of primary interest were elevated in the Camp Lejeune cohort. Of these cancers, the elevated HRs for kidney cancer, cervical cancer, leukemias and Hodgkin lymphoma occurred primarily or exclusively among those with higher cumulative exposures. The HRs for several diseases of secondary interest were also elevated. Of these diseases, the elevated HR for lung cancer occurred exclusively among those with higher cumulative exposures.

In analyses internal to the Camp Lejeune cohort, we observed monotonic trends for cumulative exposure to one or more contaminant and kidney cancer and Hodgkin lymphoma. For ALS, HRs > 1.90 were observed in the high exposure category for all the contaminants except benzene.

Drinking water studies conducted at Cape Cod, MA found associations between PCE and several cancers: lung, bladder, rectal, leukemia, and female breast [10-12]. All these cancers except bladder cancer were also elevated in comparisons between the Camp Lejeune and Camp Pendleton cohorts. In the NJ study, associations were observed for specific subgroupings of leukemia and NHL [9]. However, NHL was not elevated in our study.

Camp Pendleton did not have contaminated drinking water, but similar to Camp Lejeune, there were NPL sites located on the base. Although a public health assessment conducted by ATSDR at Camp Pendleton found “no apparent public health hazard” from these toxic waste sites [14], there was concern that the potential for exposure could not be ruled out. Therefore, we decided to compare both the Camp Lejeune and Camp Pendleton cohorts to the U.S. mortality rates. We realized that it was unlikely that any of the mortality rates at Camp Lejeune or Camp Pendleton would be elevated compared to the U.S. mortality rates because of the healthy veteran effect bias [29]. The effect of this bias is sufficiently strong to produce SMRs of ≤0.80 for cancer mortality when military personnel are compared to the U.S. population [29]. Moreover, since the median age at the end of follow-up was only 49 years, we expected that it would be too soon to observe elevations in either cohort. Nevertheless, we observed SMRs > 1.0 for three diseases of primary interest in the Camp Lejeune cohort: kidney cancer, multiple myeloma, and cervical cancer.

By the end of the study, there was one death in the Camp Lejeune cohort whose underlying cause was male breast cancer. However, many cases of male breast cancer among those who resided at Camp Lejeune have been identified in media reports and by diligent work conducted by members of the exposed population. Because male breast cancer has a relatively high survival rate, ATSDR collected data from the Veterans Affairs’s cancer registry and is currently evaluating the data regarding conducting a case-control study of male breast cancer incidence.

We conducted comparisons between Camp Lejeune and Camp Pendleton to minimize the bias due to the healthy veteran effect and because of concern that everyone at Camp Lejeune was exposed to contaminated drinking water during daily activities if not at the residence. The Camp Pendleton cohort was an appropriate comparison population. Demographics and the healthy veteran effect were similar in both cohorts. The only major difference was drinking water contamination at Camp Lejeune.

### Limitations

The study had several strengths including large cohorts, small percentage of loss to follow-up, and rigorous reconstruction of historical levels of drinking water contamination. However, there were several limitations. The average residence at Camp Lejeune was about 19 months (standard deviation = 13 months, range: 3-102 months). Many had short exposure durations that likely reduced the magnitude of the effects observed and made interpretation difficult.

A serious limitation was exposure misclassification, likely non-differential since exposure assignments should be unrelated to disease status. Such misclassification could bias HRs in comparisons between Camp Lejeune (“exposed”) and Camp Pendleton (“unexposed”) toward the null value of 1.0, resulting in underestimates of true effects of exposure. In analyses within the Camp Lejeune cohort, such bias could distort exposure-response relationships, e.g., producing non-monotonic trends that attenuate or turn negative at high exposure levels [20,34].

There were several sources of exposure misclassification. First, because historical research was necessary to identify units stationed at each base, errors in base assignment likely occurred. Second, determining a unit’s barrack location at Camp Lejeune was based primarily on recollections of retired marines. Third, family housing data inaccuracies hindered matching of married individuals to base housing, so some may have been wrongly assigned as living off-base and unexposed. Fourth, many stationed at Camp Lejeune spent time away from the base for training or deployment.

For the comparisons between the Camp Lejeune and Camp Pendleton cohorts, it is likely that the sensitivity of the exposure classification would be very high (e.g., >0.95) and the false-negative proportion would be very low because very few of those classified as “unexposed” (i.e., the Camp Pendleton cohort) would have an exposure to these contaminants. On the other hand, the specificity of the exposure classification would be much lower (e.g., between 0.70 and 0.85) because all members of the Camp Lejeune cohort were considered “exposed” although it is likely that some were not exposed. To apply a method to correct for non-differential exposure misclassification bias [35], we created a two-by-two contingency table by ignoring censoring, making the base location a time-independent variable, and forced the resulting odds ratio to be similar to the observed hazard ratio. Assuming a sensitivity of 0.98 and a specificity ranging from 0.70 to 0.85, the kidney cancer HR of 1.35 in the comparison between Camp Lejeune and Camp Pendleton could increase between 6% and 18% (i.e., the misclassification-corrected HR could increase between 1.43 and 1.59).

Disease misclassification bias (both false positives and false negatives) is also a possibility. For example, some cancers of the digestive system and oral cavity/pharynx appear to be underreported on death certificates compared to cancer registry data, whereas cancers of the esophagus, lung, liver and brain may be over reported compared to cancer registry data [36]. However, it is likely that such disease misclassification was non-differential and would tend to bias the effect measures towards the null.

Another limitation was lack of information on smoking and other risk factors. Such risk factors, if associated with exposure status, could be confounders, biasing HRs in either direction and distorting exposure-response relationships. However, both bases had similar demographics so it is unlikely that confounding was a major source of bias in comparisons between the two bases. It is also unlikely that unmeasured risk factors would be associated with contaminant cumulative exposure levels.

We evaluated smoking-related diseases not known to be associated with solvent exposure to evaluate possible confounding by smoking. In comparisons between the two cohorts, we observed an HR of 1.15 for stomach cancer suggesting that the confounding effect of smoking would be no more than 13% and within the range observed in occupational studies [37]. However, we observed very slight elevations in HRs for COPD and cardiovascular disease, and HRs below 1.00 for oral and laryngeal cancers, suggesting that the confounding effect of smoking would likely be less than 10%.

For the comparisons of cumulative exposure within Camp Lejeune, there is mixed evidence of confounding by smoking. For example, the HRs for oral cancers and stomach cancer are between 1.4 and 1.8 which would indicate the potential for considerable confounding by smoking. On the other hand, the HRs for COPD, esophageal cancer, and pancreatic cancer are all less than 1.00 indicating no confounding by smoking, and the results for lung cancer, bladder cancer and cardiovascular disease (i.e., HRs between 1.10 and 1.20) indicate that confounding by smoking would be no more than 15%. Given these results, the cumulative exposure comparisons within the Camp Lejeune cohort should be minimally affected by confounding due to smoking.

Many HR estimates lacked precision, as indicated by wide confidence intervals, due to small numbers of specific causes of death. Lack of precision in the HR estimates indicates uncertainty about the actual magnitude of the effects of the drinking water exposures on specific causes of death. Despite the large sizes of the cohorts, there were relatively small numbers of specific causes of death due to the healthy veteran effect and because most people in the cohort were younger than 55 at the end of follow-up.

## Conclusion

The study found elevated HRs in the Camp Lejeune cohort for several causes of mortality including kidney cancer, liver cancer, esophageal cancer, cervical cancer, multiple myeloma, Hodgkin lymphoma, and ALS. However, the precision of many HR estimates was low as indicated by wide confidence intervals. Approximately 97% of the Camp Lejeune cohort was under the age of 55 and less than 6% had died by the end of the study. Long-term follow-up would be necessary for a comprehensive assessment of the effects of exposures to the contaminated drinking water at the base.

## Abbreviations

ATSDR: Agency for toxic substances and disease registry; AIC: Akaike’s information criterion; ALS: Amyotrophic lateral sclerosis; COPD: Chronic obstructive pulmonary disease; CI: Confidence interval; DMF: Death master file; DMDC: Defense manpower data center; HP: Hadnot point; HR: Hazard ratio; ICD: International classification of diseases; LTAS: Life table analysis system; MCL: Maximum contaminant level; μg/L: Micrograms per liter; mg/day: Milligrams per day; MS: Multiple sclerosis; NDI: National death index; NHL: Non-Hodgkin lymphoma; NTP: National toxicology program; ORES: Office of research, evaluation and statistics; ppm: Parts per million; RCS: Restricted cubic spline functions; SSN: Social Security number; SSA: Social Security Administration; SMR: Standardized mortality ratio; TT: Tarawa Terrace; TVOC: Total amount of the contaminants; TCE: Trichloroethylene; PCE: Tetrachloroethylene or perchloroethylene; USMC: United States Marine Corps; EPA: United States Environmental Protection Agency.

## Competing interests

All authors declare they have no actual or potential competing financial interest.

## Authors’ contributions

FJB participated in the study design, data collection, analysis and interpretation of data, and drafted the manuscript. PZR participated in the study design, data collection, interpretation of data, and helped draft the manuscript. MM conducted the water modeling. TCL assisted with analysis and interpretation of data. All authors read and approved the final manuscript.

## Supplementary Material

Additional file 1: Table S3 Categorical cumulative exposures, Camp Lejeune compared to Camp Pendleton (referent). Click here for file

Additional file 2: Table S1 Categorical Cumulative Exposures and Underlying Cause of Death. Click here for file

Additional file 3: Table S2 Cumulative Exposures and Underlying Cause of Death. Click here for file

Additional file 4: Figures S1-S3 Splines of selected causes of death and cumulative exposures. Click here for file
