# Supplementary material for: Evaluation of mortality among marines and navy personnel exposed to contaminated drinking water at USMC base Camp Lejeune: a retrospective cohort study
Source: Environ Health. 2014 Feb 19;13:10. doi: 10.1186/1476-069X-13-10 (PMC3943370; doi:10.1186/1476-069X-13-10)
Supplement: Additional file 1: Table S3 — Categorical cumulative exposures, Camp Lejeune compared to Camp Pendleton (referent). [file 1476-069X-13-10-S1.docx]

**Additional file 1: Table S3**: Categorized cumulative exposure, Camp Lejeune compared to Camp Pendleton (referent), 10 year lag, adjusted hazard ratios (95% CI).

**Total Contaminants (TVOC)**

| **Underlying Cause of Death** | No/very low cumulative exposure  HR LCL UCL | | | Low to high cumulative exposure  HR LCL UCL | | |
| --- | --- | --- | --- | --- | --- | --- |
| All Cancers | 1.09 | 0.97 | 1.22 | 1.11 | 1.01 | 1.23 |
| **Diseases of Primary Interest:** | | | | | | |
| Kidney Cancer | 0.92 | 0.46 | 1.85 | 1.50 | 0.91 | 2.47 |
| Bladder Cancer | 0.64 | 0.18 | 2.28 | 0.94 | 0.39 | 2.25 |
| Liver Cancer | 1.29 | 0.73 | 2.30 | 1.35 | 0.84 | 2.16 |
| Esophageal Cancer | 1.71 | 0.89 | 3.30 | 1.26 | 0.71 | 2.24 |
| Cervical Cancer | 1.12 | 0.19 | 6.73 | 5.84 | 0.81 | 42.23 |
| Hematopoietic Cancers | 0.81 | 0.60 | 1.09 | 1.18 | 0.91 | 1.52 |
| Multiple Myeloma | 1.13 | 0.38 | 3.31 | 1.70 | 0.75 | 3.86 |
| Leukemia | 0.69 | 0.42 | 1.13 | 1.45 | 0.97 | 2.17 |
| Non-Hodgkin Lymphoma | 0.93 | 0.59 | 1.49 | 0.80 | 0.52 | 1.22 |
| Hodgkin Lymphoma | 0.72 | 0.34 | 1.50 | 1.67 | 0.80 | 3.50 |
| **Diseases of Secondary Interest:** | | | | | | |
| Pancreatic Cancer | 1.71 | 1.03 | 2.84 | 1.14 | 0.72 | 1.79 |
| Colorectal Cancers | 1.44 | 1.00 | 2.07 | 1.14 | 0.82 | 1.57 |
| Rectal Cancer | 1.62 | 0.69 | 3.82 | 1.54 | 0.77 | 3.10 |
| Colon Cancer | 1.40 | 0.94 | 2.09 | 1.05 | 0.72 | 1.51 |
| Lung Cancer | 1.04 | 0.79 | 1.36 | 1.22 | 0.99 | 1.49 |
| Brain Cancer | 1.03 | 0.68 | 1.57 | 0.93 | 0.64 | 1.35 |
| Soft-tissue Cancers | 1.66 | 0.85 | 3.25 | 1.17 | 0.57 | 2.37 |
| Oral Cancers | 0.71 | 0.34 | 1.51 | 0.84 | 0.47 | 1.52 |
| Breast Cancer | 0.97 | 0.35 | 2.69 | 1.55 | 0.31 | 7.67 |
| Prostate Cancer | 1.46 | 0.58 | 3.64 | 1.08 | 0.49 | 2.38 |
| Multiple Sclerosis | 1.59 | 0.56 | 4.54 | 0.97 | 0.35 | 2.69 |
| ALS | 0.56 | 0.21 | 1.47 | 0.92 | 0.49 | 1.71 |
| Kidney Disease | 0.74 | 0.36 | 1.51 | 1.12 | 0.68 | 1.86 |
| Liver Disease | 0.84 | 0.63 | 1.11 | 0.87 | 0.70 | 1.08 |
| **Smoking-related Diseases** (not known to be related to solvent exposure): | | | | | | |
| Stomach Cancer | 1.16 | 0.58 | 2.34 | 1.26 | 0.73 | 2.17 |
| COPD | 1.19 | 0.69 | 2.06 | 1.00 | 0.62 | 1.62 |
| Cardiovascular | 1.00 | 0.90 | 1.11 | 1.06 | 0.98 | 1.15 |

**Benzene**

| **Underlying Cause of Death** | No/very low cumulative exposure  HR LCL UCL | | | Low to high cumulative exposure  HR LCL UCL | | |
| --- | --- | --- | --- | --- | --- | --- |
| All Cancers | 1.07 | 0.95 | 1.19 | 1.13 | 1.02 | 1.25 |
| **Diseases of Primary Interest:** | | | | | | |
| Kidney Cancer | 1.03 | 0.54 | 1.94 | 1.48 | 0.88 | 2.47 |
| Bladder Cancer | 0.52 | 0.15 | 1.85 | 1.07 | 0.44 | 2.60 |
| Liver Cancer | 1.25 | 0.72 | 2.17 | 1.39 | 0.86 | 2.24 |
| Esophageal Cancer | 1.48 | 0.77 | 2.85 | 1.37 | 0.77 | 2.43 |
| Cervical Cancer | 1.08 | 0.18 | 6.50 | 7.51 | 1.03 | 54.53 |
| Hematopoietic Cancers | 0.83 | 0.62 | 1.10 | 1.19 | 0.92 | 1.55 |
| Multiple Myeloma | 1.37 | 0.53 | 3.58 | 1.57 | 0.66 | 3.71 |
| Leukemia | 0.73 | 0.46 | 1.16 | 1.47 | 0.97 | 2.23 |
| Non-Hodgkin Lymphoma | 0.92 | 0.59 | 1.45 | 0.80 | 0.52 | 1.23 |
| Hodgkin Lymphoma | 0.68 | 0.33 | 1.42 | 1.87 | 0.89 | 3.95 |
| **Diseases of Secondary Interest:** | | | | | | |
| Pancreatic Cancer | 1.56 | 0.95 | 2.56 | 1.18 | 0.74 | 1.87 |
| Colorectal Cancers | 1.41 | 1.00 | 2.00 | 1.13 | 0.80 | 1.57 |
| Rectal Cancer | 1.51 | 0.66 | 3.45 | 1.61 | 0.78 | 3.28 |
| Colon Cancer | 1.39 | 0.95 | 2.04 | 1.02 | 0.70 | 1.49 |
| Lung Cancer | 1.04 | 0.80 | 1.34 | 1.23 | 1.00 | 1.52 |
| Brain Cancer | 0.97 | 0.64 | 1.46 | 0.97 | 0.66 | 1.43 |
| Soft-tissue Cancers | 1.51 | 0.77 | 2.95 | 1.28 | 0.63 | 2.60 |
| Oral Cancers | 0.71 | 0.34 | 1.45 | 0.86 | 0.47 | 1.57 |
| Breast Cancer | 0.94 | 0.34 | 2.61 | 1.89 | 0.38 | 9.33 |
| Prostate Cancer | 1.44 | 0.60 | 3.44 | 1.07 | 0.47 | 2.39 |
| Multiple Sclerosis | 1.72 | 0.64 | 4.64 | 0.85 | 0.29 | 2.50 |
| ALS | 0.76 | 0.34 | 1.68 | 0.83 | 0.42 | 1.61 |
| Kidney Disease | 0.79 | 0.41 | 1.54 | 1.12 | 0.67 | 1.87 |
| Liver Disease | 0.86 | 0.65 | 1.12 | 0.86 | 0.69 | 1.08 |
| **Smoking-related Diseases** (not known to be related to solvent exposure): | | | | | | |
| Stomach Cancer | 1.45 | 0.78 | 2.69 | 1.08 | 0.60 | 1.94 |
| COPD | 1.19 | 0.71 | 2.01 | 0.99 | 0.60 | 1.62 |
| Cardiovascular | 1.01 | 0.91 | 1.12 | 1.06 | 0.97 | 1.15 |

**Vinyl Chloride**

| **Underlying Cause of Death** | No/very low cumulative exposure  HR LCL UCL | | | Low to high cumulative exposure  HR LCL UCL | | |
| --- | --- | --- | --- | --- | --- | --- |
| All Cancers | 1.07 | 0.95 | 1.20 | 1.13 | 1.02 | 1.25 |
| **Diseases of Primary Interest:** | | | | | | |
| Kidney Cancer | 0.91 | 0.46 | 1.78 | 1.55 | 0.94 | 2.57 |
| Bladder Cancer | 0.75 | 0.24 | 2.34 | 0.88 | 0.35 | 2.22 |
| Liver Cancer | 1.33 | 0.77 | 2.32 | 1.33 | 0.82 | 2.14 |
| Esophageal Cancer | 1.70 | 0.89 | 3.22 | 1.25 | 0.70 | 2.24 |
| Cervical Cancer | 1.11 | 0.19 | 6.70 | 5.99 | 0.83 | 43.35 |
| Hematopoietic Cancers | 0.83 | 0.63 | 1.10 | 1.18 | 0.91 | 1.54 |
| Multiple Myeloma | 1.23 | 0.45 | 3.37 | 1.67 | 0.72 | 3.87 |
| Leukemia | 0.80 | 0.51 | 1.26 | 1.37 | 0.90 | 2.08 |
| Non-Hodgkin Lymphoma | 0.88 | 0.56 | 1.39 | 0.83 | 0.54 | 1.28 |
| Hodgkin Lymphoma | 0.68 | 0.33 | 1.42 | 1.86 | 0.89 | 3.92 |
| **Diseases of Secondary Interest:** | | | | | | |
| Pancreatic Cancer | 1.72 | 1.06 | 2.80 | 1.10 | 0.69 | 1.75 |
| Colorectal Cancers | 1.41 | 0.99 | 2.01 | 1.13 | 0.81 | 1.58 |
| Rectal Cancer | 1.59 | 0.70 | 3.65 | 1.55 | 0.76 | 3.16 |
| Colon Cancer | 1.37 | 0.93 | 2.03 | 1.04 | 0.72 | 1.52 |
| Lung Cancer | 1.00 | 0.77 | 1.31 | 1.24 | 1.01 | 1.53 |
| Brain Cancer | 0.92 | 0.60 | 1.39 | 1.02 | 0.69 | 1.48 |
| Soft-tissue Cancers | 1.64 | 0.85 | 3.16 | 1.15 | 0.56 | 2.38 |
| Oral Cancers | 0.65 | 0.31 | 1.38 | 0.89 | 0.50 | 1.62 |
| Breast Cancer | 0.97 | 0.35 | 2.68 | 1.58 | 0.32 | 7.82 |
| Prostate Cancer | 1.31 | 0.52 | 3.28 | 1.14 | 0.52 | 2.51 |
| Multiple Sclerosis | 1.47 | 0.52 | 4.18 | 1.02 | 0.37 | 2.83 |
| ALS | 0.59 | 0.24 | 1.45 | 0.92 | 0.49 | 1.75 |
| Kidney Disease | 0.75 | 0.38 | 1.49 | 1.14 | 0.68 | 1.89 |
| Liver Disease | 0.85 | 0.64 | 1.11 | 0.87 | 0.70 | 1.08 |
| **Smoking-related Diseases** (not known to be related to solvent exposure): | | | | | | |
| Stomach Cancer | 1.31 | 0.69 | 2.50 | 1.17 | 0.66 | 2.07 |
| COPD | 1.18 | 0.69 | 2.02 | 1.00 | 0.61 | 1.63 |
| Cardiovascular | 1.01 | 0.91 | 1.12 | 1.06 | 0.97 | 1.15 |

**Trichloroethylene (TCE)**

| **Underlying Cause of Death** | No/very low cumulative exposure  HR LCL UCL | | | Low to high cumulative exposure  HR LCL UCL | | |
| --- | --- | --- | --- | --- | --- | --- |
| All Cancers | 1.08 | 0.96 | 1.21 | 1.12 | 1.01 | 1.24 |
| **Diseases of Primary Interest:** | | | | | | |
| Kidney Cancer | 0.95 | 0.48 | 1.86 | 1.50 | 0.91 | 2.49 |
| Bladder Cancer | 0.81 | 0.26 | 2.50 | 0.84 | 0.34 | 2.10 |
| Liver Cancer | 1.30 | 0.74 | 2.29 | 1.34 | 0.84 | 2.16 |
| Esophageal Cancer | 1.74 | 0.92 | 3.30 | 1.24 | 0.69 | 2.21 |
| Cervical Cancer | 1.12 | 0.19 | 6.73 | 5.85 | 0.81 | 42.29 |
| Hematopoietic Cancers | 0.83 | 0.62 | 1.10 | 1.18 | 0.91 | 1.53 |
| Multiple Myeloma | 1.31 | 0.48 | 3.59 | 1.59 | 0.69 | 3.67 |
| Leukemia | 0.76 | 0.47 | 1.21 | 1.40 | 0.93 | 2.12 |
| Non-Hodgkin Lymphoma | 0.90 | 0.57 | 1.41 | 0.82 | 0.53 | 1.26 |
| Hodgkin Lymphoma | 0.69 | 0.33 | 1.45 | 1.79 | 0.85 | 3.74 |
| **Diseases of Secondary Interest:** | | | | | | |
| Pancreatic Cancer | 1.80 | 1.11 | 2.92 | 1.07 | 0.67 | 1.71 |
| Colorectal Cancers | 1.40 | 0.98 | 2.01 | 1.15 | 0.83 | 1.59 |
| Rectal Cancer | 1.51 | 0.64 | 3.56 | 1.60 | 0.79 | 3.21 |
| Colon Cancer | 1.38 | 0.93 | 2.05 | 1.04 | 0.72 | 1.52 |
| Lung Cancer | 1.02 | 0.78 | 1.34 | 1.23 | 1.00 | 1.51 |
| Brain Cancer | 0.96 | 0.63 | 1.45 | 0.98 | 0.67 | 1.43 |
| Soft-tissue Cancers | 1.67 | 0.86 | 3.22 | 1.13 | 0.55 | 2.34 |
| Oral Cancers | 0.68 | 0.32 | 1.43 | 0.87 | 0.48 | 1.58 |
| Breast Cancer | 0.97 | 0.35 | 2.69 | 1.56 | 0.32 | 7.69 |
| Prostate Cancer | 1.36 | 0.54 | 3.39 | 1.12 | 0.51 | 2.46 |
| Multiple Sclerosis | 1.49 | 0.52 | 4.22 | 1.01 | 0.36 | 2.81 |
| ALS | 0.63 | 0.26 | 1.54 | 0.89 | 0.47 | 1.69 |
| Kidney Disease | 0.77 | 0.39 | 1.53 | 1.12 | 0.67 | 1.86 |
| Liver Disease | 0.82 | 0.62 | 1.08 | 0.88 | 0.71 | 1.10 |
| **Smoking-related Diseases** (not known to be related to solvent exposure): | | | | | | |
| Stomach Cancer | 1.17 | 0.59 | 2.32 | 1.25 | 0.72 | 2.17 |
| COPD | 1.20 | 0.70 | 2.05 | 0.99 | 0.61 | 1.62 |
| Cardiovascular | 1.00 | 0.91 | 1.11 | 1.06 | 0.97 | 1.15 |

**Tetrachloroethylene (perchloroethylene, PCE)**

| **Underlying Cause of Death** | No/very low cumulative exposure  HR LCL UCL | | | Low to high cumulative exposure  HR LCL UCL | | |
| --- | --- | --- | --- | --- | --- | --- |
| All Cancers | 1.07 | 0.95 | 1.20 | 1.13 | 1.02 | 1.25 |
| **Diseases of Primary Interest:** | | | | | | |
| Kidney Cancer | 0.91 | 0.46 | 1.78 | 1.55 | 0.94 | 2.57 |
| Bladder Cancer | 0.75 | 0.24 | 2.33 | 0.89 | 0.35 | 2.22 |
| Liver Cancer | 1.33 | 0.76 | 2.31 | 1.33 | 0.82 | 2.14 |
| Esophageal Cancer | 1.69 | 0.89 | 3.21 | 1.25 | 0.70 | 2.24 |
| Cervical Cancer | 1.11 | 0.18 | 6.69 | 6.04 | 0.84 | 43.73 |
| Hematopoietic Cancers | 0.83 | 0.63 | 1.10 | 1.19 | 0.91 | 1.54 |
| Multiple Myeloma | 1.23 | 0.45 | 3.36 | 1.67 | 0.72 | 3.87 |
| Leukemia | 0.80 | 0.51 | 1.26 | 1.37 | 0.90 | 2.08 |
| Non-Hodgkin Lymphoma | 0.88 | 0.56 | 1.38 | 0.83 | 0.54 | 1.28 |
| Hodgkin Lymphoma | 0.68 | 0.33 | 1.42 | 1.87 | 0.89 | 3.92 |
| **Diseases of Secondary Interest:** | | | | | | |
| Pancreatic Cancer | 1.72 | 1.06 | 2.80 | 1.10 | 0.69 | 1.76 |
| Colorectal Cancers | 1.41 | 0.99 | 2.01 | 1.14 | 0.82 | 1.58 |
| Rectal Cancer | 1.59 | 0.69 | 3.64 | 1.55 | 0.76 | 3.17 |
| Colon Cancer | 1.37 | 0.92 | 2.03 | 1.04 | 0.72 | 1.52 |
| Lung Cancer | 1.00 | 0.77 | 1.31 | 1.25 | 1.01 | 1.53 |
| Brain Cancer | 0.92 | 0.60 | 1.39 | 1.02 | 0.70 | 1.49 |
| Soft-tissue Cancers | 1.64 | 0.85 | 3.16 | 1.15 | 0.56 | 2.39 |
| Oral Cancers | 0.65 | 0.31 | 1.38 | 0.90 | 0.50 | 1.62 |
| Breast Cancer | 0.96 | 0.35 | 2.68 | 1.59 | 0.32 | 7.86 |
| Prostate Cancer | 1.31 | 0.52 | 3.27 | 1.15 | 0.52 | 2.52 |
| Multiple Sclerosis | 1.47 | 0.52 | 4.17 | 1.02 | 0.37 | 2.83 |
| ALS | 0.59 | 0.24 | 1.45 | 0.93 | 0.49 | 1.75 |
| Kidney Disease | 0.75 | 0.38 | 1.49 | 1.14 | 0.68 | 1.89 |
| Liver Disease | 0.84 | 0.64 | 1.11 | 0.87 | 0.70 | 1.08 |
| **Smoking-related Diseases** (not known to be related to solvent exposure): | | | | | | |
| Stomach Cancer | 1.30 | 0.68 | 2.49 | 1.17 | 0.67 | 2.07 |
| COPD | 1.18 | 0.69 | 2.02 | 1.00 | 0.61 | 1.63 |
| Cardiovascular | 1.02 | 0.92 | 1.12 | 1.05 | 0.97 | 1.15 |
