# Supplementary material for: Evaluation of mortality among marines and navy personnel exposed to contaminated drinking water at USMC base Camp Lejeune: a retrospective cohort study
Source: Environ Health. 2014 Feb 19;13:10. doi: 10.1186/1476-069X-13-10 (PMC3943370; doi:10.1186/1476-069X-13-10)
Supplement: Additional file 2: Table S1 — Categorical Cumulative Exposures and Underlying Cause of Death. [file 1476-069X-13-10-S2.docx]

**Additional file 2: Table S1: Categorical Cumulative Exposures and Underlying Cause of Death**

Cumulative Exposure to **Total Volatile Organic Compounds** (TVOC): PCE, TCE, DCE, VC, and benzene. (N = 154,932). Reference group has no exposure to TVOC. Hazard Ratio, Adjusted, 10 year exposure lag.

| **Underlying**  **Cause of Death** | Low Exposure  **HR** LCL UCL N | | | | Medium Exposure  **HR** LCL UCL N | | | | High Exposure  **HR** LCL UCL N | | | |
| --- | --- | --- | --- | --- | --- | --- | --- | --- | --- | --- | --- | --- |
| All cancers (N=1,078) | **1.01** | *0.85* | *1.20* | 224 | **1.01** | *0.84* | *1.20* | 218 | **1.01** | *0.85* | *1.20* | 220 |
| **Diseases of Primary Interest** | | | | | | | | | | | | |
| **Underlying**  **Cause of Death** | Low Exposure  **HR** LCL UCL N | | | | Medium Exposure  **HR** LCL UCL N | | | | High Exposure  **HR** LCL UCL N | | | |
| Kidney Cancer (N=42) | **1.42** | *0.58* | *3.47* | 10 | **1.44** | *0.58* | *3.59* | 10 | **1.54** | *0.63* | *3.75* | 11 |
| Bladder Cancer (N=11) | **0.63** | *0.06* | *6.93* | 1 | **3.33** | *0.64* | *17.37* | 5 | **1.20** | *0.17* | *8.61* | 2 |
| Liver Cancer (N=51) | **0.95** | *0.43* | *2.06* | 11 | **1.14** | *0.53* | *2.44* | 13 | **0.85** | *0.37* | *1.98* | 9 |
| Esophageal Cancer (N=35) | **1.01** | *0.43* | *2.37* | 9 | **0.51** | *0.18* | *1.45* | 5 | **0.73** | *0.29* | *1.86* | 7 |
| Hematopoietic Cancers (N=165) | **1.53** | *0.99* | *2.36* | 40 | **1.03** | *0.62* | *1.70* | 25 | **1.45** | *0.91* | *2.30* | 35 |
| Multiple Myeloma (N=17) | **2.68** | *0.80* | *8.90* | 8 | **1.36** | *0.34* | *5.53* | 4 | **0.00** | *0.00* |  | 0 |
| Leukemia (N=66) | **2.50** | *1.24* | *5.03* | 19 | **1.33** | *0.56* | *3.14* | 9 | **2.33** | *1.08* | *5.03* | 15 |
| Non-Hodgkin Lymphoma (N=58) | **0.87** | *0.41* | *1.83* | 11 | **0.58** | *0.24* | *1.38* | 7 | **1.15** | *0.57* | *2.32* | 14 |
| Hodgkin (N=24) | **0.66** | *0.13* | *3.39* | 2 | **1.77** | *0.50* | *6.25* | 5 | **2.17** | *0.63* | *7.50* | 6 |
| **Diseases of Secondary Interest** | | | | | | | | | | | | |
| **Underlying**  **Cause of Death** | Low Exposure  **HR** LCL UCL N | | | | Medium Exposure  **HR** LCL UCL N | | | | High Exposure  **HR** LCL UCL N | | | |
| Pancreatic Cancer (N=57) | **0.64** | *0.31* | *1.32* | 11 | **0.72** | *0.36* | *1.46* | 12 | **0.57** | *0.27* | *1.21* | 10 |
| Colorectal Cancers (N=110) | **0.85** | *0.50* | *1.44* | 22 | **0.75** | *0.42* | *1.34* | 18 | **1.00** | *0.59* | *1.70* | 24 |
| Rectal Cancer (N=24) | **1.14** | *0.41* | *3.14* | 7 | **0.50** | *0.13* | *1.92* | 3 | **1.03** | *0.35* | *3.03* | 6 |
| Colon Cancer (N=86) | **0.76** | *0.40* | *1.42* | 15 | **0.83** | *0.44* | *1.58* | 15 | **0.99** | *0.53* | *1.82* | 18 |
| Lung Cancer (N=237) | **1.25** | *0.87* | *1.78* | 57 | **1.11** | *0.77* | *1.61* | 54 | **1.17** | *0.81* | *1.69* | 55 |
| Brain Cancer (N=74) | **0.77** | *0.39* | *1.54* | 12 | **1.08** | *0.57* | *2.06* | 16 | **0.89** | *0.45* | *1.75* | 14 |
| Soft-tissue Cancers (N=29) | **0.64** | *0.20* | *2.09* | 4 | **1.28** | *0.47* | *3.51* | 7 | **0.36** | *0.08* | *1.71* | 2 |
| Oral cancers (N=26) | **1.72** | *0.61* | *4.89* | 8 | **0.43** | *0.09* | *2.11* | 2 | **1.56** | *0.53* | *4.66* | 7 |
| Breast Cancer (female) (N=10) | **2.12** | *0.41* | *10.94* | 2 | **0.00** | *0.00* |  | 0 | **0.00** | *0.00* |  | 0 |
| Cumulative Exposure to **Total Volatile Organic Compounds** (TVOC): PCE, TCE, DCE, VC, and benzene. Reference group has no exposure to TVOC. | | | | | | | | | | | | |
| **Underlying**  **Cause of Death** | Low Exposure  **HR** LCL UCL N | | | | Medium Exposure  **HR** LCL UCL N | | | | High Exposure  **HR** LCL UCL N | | | |
| Prostate Cancer (N=18) | **0.80** | *0.23* | *2.84* | 4 | **0.72** | *0.20* | *2.58* | 4 | **0.55** | *0.14* | *2.21* | 3 |
| Multiple Sclerosis (N=12) | **0.29** | *0.03* | *2.52* | 1 | **1.13** | *0.30* | *4.27* | 4 | **0.28** | *0.03* | *2.42* | 1 |
| ALS (N=21) | **1.27** | *0.37* | *4.41* | 5 | **0.89** | *0.21* | *3.82* | 3 | **2.11** | *0.67* | *6.68* | 8 |
| Kidney Disease (N=37) | **1.64** | *0.68* | *3.94* | 11 | **1.25** | *0.50* | *3.16* | 9 | **1.01** | *0.37* | *2.74* | 7 |
| Liver Disease (N=191) | **0.86** | *0.56* | *1.32* | 33 | **1.23** | *0.83* | *1.81* | 50 | **1.15** | *0.78* | *1.72* | 46 |
| **Smoking-related Diseases** (not known to be related to solvent exposure) | | | | | | | | | | | | |
| **Underlying**  **Cause of Death** | Low Exposure  **HR** LCL UCL N | | | | Medium Exposure  **HR** LCL UCL N | | | | High Exposure  **HR** LCL UCL N | | | |
| Stomach Cancer (N=35) | **1.38** | *0.55* | *3.48* | 9 | **1.05** | *0.37* | *3.01* | 6 | **1.46** | *0.56* | *3.75* | 9 |
| COPD (N=47) | **1.12** | *0.52* | *2.40* | 12 | **0.91** | *0.42* | *2.01* | 11 | **0.33** | *0.11* | *1.01* | 4 |
| Cardiovascular (N=1,391) | **1.05** | *0.91* | *1.23* | 295 | **1.06** | *0.91* | *1.24* | 300 | **1.12** | *0.97* | *1.30* | 324 |

| **Cumulative Exposure to Benzene. Reference group has no exposure to Benzene** | | | | | | | | | | | | |
| --- | --- | --- | --- | --- | --- | --- | --- | --- | --- | --- | --- | --- |
| **Underlying**  **Cause of Death** | Low Exposure  **HR** LCL UCL N | | | | Medium Exposure  **HR** LCL UCL N | | | | High Exposure  **HR** LCL UCL N | | | |
| All cancers (N=1,078) | **1.12** | *0.94* | *1.33* | 201 | **1.06** | *0.90* | *1.26* | 220 | **0.96** | *0.80* | *1.15* | 196 |
| **Diseases of Primary Interest** | | | | | | | | | | | | |
| **Underlying**  **Cause of Death** | Low Exposure  **HR** LCL UCL N | | | | Medium Exposure  **HR** LCL UCL N | | | | High Exposure  **HR** LCL UCL N | | | |
| Kidney Cancer (N=42) | **1.31** | *0.52* | *3.29* | 8 | **1.38** | *0.58* | *3.28* | 10 | **1.36** | *0.57* | *3.25* | 10 |
| Bladder Cancer (N=11) | **0.00** | *0.00* |  | 0 | **4.04** | *0.77* | *21.18* | 5 | **2.26** | *0.37* | *13.78* | 3 |
| Liver Cancer (N=51) | **1.32** | *0.62* | *2.84* | 12 | **1.11** | *0.50* | *2.43* | 11 | **0.89** | *0.37* | *2.13* | 8 |
| Esophageal Cancer (N=35) | **1.22** | *0.52* | *2.88* | 9 | **0.87** | *0.34* | *2.19* | 7 | **0.64** | *0.23* | *1.82* | 5 |
| Hematopoietic Cancers (N=165) | **1.48** | *0.94* | *2.33* | 32 | **1.26** | *0.80* | *1.99* | 32 | **1.16** | *0.72* | *1.86* | 29 |
| Multiple Myeloma (N=17) | **1.81** | *0.53* | *6.23* | 5 | **1.47** | *0.43* | *5.05* | 5 | **0.00** | *0.00* |  | 0 |
| Leukemia (N=66) | **2.54** | *1.27* | *5.08* | 17 | **1.46** | *0.66* | *3.20* | 11 | **1.69** | *0.77* | *3.67* | 12 |
| Non-Hodgkin Lymphoma (N=58) | **0.71** | *0.30* | *1.67* | 7 | **0.91** | *0.44* | *1.91* | 11 | **0.99** | *0.48* | *2.04* | 12 |
| Hodgkin (N=24) | **1.24** | *0.30* | *5.11* | 3 | **1.88** | *0.54* | *6.61* | 5 | **1.94** | *0.54* | *6.95* | 5 |
| **Diseases of Secondary Interest** | | | | | | | | | | | | |
| **Underlying**  **Cause of Death** | Low Exposure  **HR** LCL UCL N | | | | Medium Exposure  **HR** LCL UCL N | | | | High Exposure  **HR** LCL UCL N | | | |
| Pancreatic Cancer (N=57) | **0.65** | *0.29* | *1.46* | 8 | **0.94** | *0.48* | *1.84* | 14 | **0.58** | *0.27* | *1.27* | 9 |
| Colorectal Cancers (N=110) | **0.86** | *0.48* | *1.53* | 17 | **0.83** | *0.48* | *1.46* | 19 | **0.97** | *0.57* | *1.66* | 22 |
| Rectal Cancer (N=24) | **1.38** | *0.46* | *4.11* | 6 | **0.80** | *0.23* | *2.71* | 4 | **1.00** | *0.32* | *3.16* | 5 |
| Colon Cancer (N=86) | **0.72** | *0.36* | *1.44* | 11 | **0.85** | *0.45* | *1.59* | 15 | **0.97** | *0.53* | *1.78* | 17 |
| Lung Cancer (N=237) | **1.36** | *0.96* | *1.93* | 58 | **0.98** | *0.68* | *1.43* | 46 | **1.16** | *0.81* | *1.68* | 51 |
| Brain Cancer (N=74) | **0.93** | *0.45* | *1.88* | 11 | **1.12** | *0.60* | *2.12* | 16 | **0.87** | *0.44* | *1.72* | 13 |
| Soft-tissue Cancers (N=29) | **0.96** | *0.29* | *3.13* | 4 | **1.67** | *0.63* | *4.42* | 8 | **0.22** | *0.03* | *1.74* | 1 |
| Oral cancers (N=26) | **2.07** | *0.73* | *5.85* | 8 | **0.70** | *0.18* | *2.76* | 3 | **1.28** | *0.40* | *4.14* | 5 |
| Prostate Cancer (N=18) | **1.10** | *0.35* | *3.50* | 5 | **0.38** | *0.08* | *1.84* | 2 | **0.60** | *0.16* | *2.35* | 3 |
| Breast Cancer (female) (N=10) | **2.21** | *0.43* | *11.45* | 2 | **0.00** | *0.00* |  | 0 | **0.00** | *0.00* |  | 0 |
| Multiple Sclerosis (N=12) | **0.00** | *0.00* |  | 0 | **1.04** | *0.29* | *3.71* | 4 | **0.26** | *0.03* | *2.16* | 1 |
| Cumulative Exposure to **Benzene.** Reference group has no exposure to Benzene | | | | | | | | | | | | |
| **Underlying**  **Cause of Death** | Low Exposure  **HR** LCL UCL N | | | | Medium Exposure  **HR** LCL UCL N | | | | High Exposure  **HR** LCL UCL N | | | |
| ALS (N=21) | **0.29** | *0.04* | *2.42* | 1 | **1.19** | *0.37* | *3.85* | 5 | **1.60** | *0.54* | *4.70* | 7 |
| Kidney Disease (N=37) | **1.69** | *0.73* | *3.89* | 11 | **1.21** | *0.50* | *2.93* | 9 | **0.69** | *0.24* | *2.00* | 5 |
| Liver Disease (N=191) | **0.92** | *0.60* | *1.40* | 33 | **1.09** | *0.74* | *1.60* | 44 | **1.10** | *0.74* | *1.63* | 43 |
| **Smoking-related Diseases** (not known to be related to solvent exposure) | | | | | | | | | | | | |
| **Underlying**  **Cause of Death** | Low Exposure  **HR** LCL UCL N | | | | Medium Exposure  **HR** LCL UCL N | | | | High Exposure  **HR** LCL UCL N | | | |
| Stomach Cancer (N=35) | **0.51** | *0.14* | *1.80* | 3 | **0.54** | *0.17* | *1.69* | 4 | **1.53** | *0.68* | *3.46* | 12 |
| COPD (N=47) | **1.32** | *0.64* | *2.74* | 13 | **0.61** | *0.25* | *1.48* | 7 | **0.44** | *0.16* | *1.20* | 5 |
| Cardiovascular (N=1,391) | **1.09** | *0.94* | *1.28* | 260 | **1.03** | *0.88* | *1.19* | 288 | **1.07** | *0.92* | *1.24* | 303 |

| **Cumulative Exposure to Vinyl Chloride. Reference group has no exposure to vinyl chloride** | | | | | | | | | | | | |
| --- | --- | --- | --- | --- | --- | --- | --- | --- | --- | --- | --- | --- |
| **Underlying**  **Cause of Death** | Low Exposure  **HR** LCL UCL N | | | | Medium Exposure  **HR** LCL UCL N | | | | High Exposure  **HR** LCL UCL N | | | |
| All cancers (N=1,078) | **1.08** | *0.91* | *1.28* | 214 | **1.05** | *0.88* | *1.25* | 210 | **0.99** | *0.83* | *1.19* | 206 |
| **Diseases of Primary Interest** | | | | | | | | | | | | |
| **Underlying**  **Cause of Death** | Low Exposure  **HR** LCL UCL N | | | | Medium Exposure  **HR** LCL UCL N | | | | High Exposure  **HR** LCL UCL N | | | |
| Kidney Cancer (N=42) | **1.66** | *0.68* | *4.04* | 10 | **1.61** | *0.65* | *3.98* | 10 | **1.51** | *0.61* | *3.74* | 10 |
| Bladder Cancer (N=11) | **0.00** | *0.00* |  | 0 | **2.59** | *0.61* | *10.98* | 5 | **0.91** | *0.15* | *5.52* | 2 |
| Liver Cancer (N=51) | **1.01** | *0.47* | *2.19* | 11 | **1.03** | *0.47* | *2.25* | 11 | **0.89** | *0.39* | *2.06* | 9 |
| Esophageal Cancer (N=35) | **1.10** | *0.49* | *2.51* | 10 | **0.55** | *0.19* | *1.53* | 5 | **0.54** | *0.19* | *1.52* | 5 |
| Hematopoietic Cancers (N=165) | **1.57** | *1.02* | *2.42* | 37 | **1.06** | *0.65* | *1.73* | 25 | **1.24** | *0.78* | *1.98* | 31 |
| Multiple Myeloma (N=17) | **2.49** | *0.77* | *8.00* | 7 | **1.38** | *0.36* | *5.31* | 4 | **0.00** | *0.00* |  | 0 |
| Leukemia (N=66) | **1.85** | *0.93* | *3.68* | 15 | **1.27** | *0.58* | *2.80* | 10 | **1.63** | *0.78* | *3.43* | 13 |
| Non-Hodgkin Lymphoma (N=58) | **1.19** | *0.58* | *2.43* | 12 | **0.57** | *0.23* | *1.42* | 6 | **1.15** | *0.56* | *2.35* | 13 |
| Hodgkin (N=24) | **1.20** | *0.29* | *4.94* | 3 | **2.07** | *0.59* | *7.27* | 5 | **1.99** | *0.56* | *7.13* | 5 |
| **Diseases of Secondary Interest** | | | | | | | | | | | | |
| **Underlying**  **Cause of Death** | Low Exposure  **HR** LCL UCL N | | | | Medium Exposure  **HR** LCL UCL N | | | | High Exposure  **HR** LCL UCL N | | | |
| Pancreatic Cancer (N=57) | **0.65** | *0.31* | *1.36* | 10 | **0.63** | *0.30* | *1.32* | 10 | **0.58** | *0.28* | *1.22* | 10 |
| Colorectal Cancers (N=110) | **0.86** | *0.50* | *1.50* | 19 | **0.73** | *0.40* | *1.33* | 16 | **1.10** | *0.65* | *1.85* | 25 |
| Rectal Cancer (N=24) | **1.14** | *0.40* | *3.29* | 6 | **0.56** | *0.15* | *2.14* | 3 | **1.10** | *0.38* | *3.23* | 6 |
| Colon Cancer (N=86) | **0.78** | *0.40* | *1.50* | 13 | **0.79** | *0.40* | *1.53* | 13 | **1.10** | *0.61* | *2.00* | 19 |
| Lung Cancer (N=237) | **1.30** | *0.91* | *1.85* | 58 | **1.15** | *0.79* | *1.66* | 51 | **1.21** | *0.84* | *1.75* | 53 |
| Brain Cancer (N=74) | **1.00** | *0.50* | *2.00* | 12 | **1.39** | *0.74* | *2.61* | 17 | **0.95** | *0.47* | *1.89* | 13 |
| Soft-tissue Cancers (N=29) | **0.76** | *0.24* | *2.45* | 4 | **1.17** | *0.42* | *3.30* | 6 | **0.38** | *0.08* | *1.76* | 2 |
| Oral cancers (N=26) | **2.06** | *0.71* | *5.96* | 8 | **0.78** | *0.19* | *3.17* | 3 | **1.62** | *0.51* | *5.12* | 6 |
| Prostate Cancer (N=18) | **0.88** | *0.25* | *3.14* | 4 | **0.84** | *0.23* | *2.99* | 4 | **0.63** | *0.16* | *2.54* | 3 |
| Breast Cancer (female) (N=10) | **1.83** | *0.35* | *9.43* | 2 | **0.00** | *0.00* |  | 0 | **0.00** | *0.00* |  | 0 |
| Multiple Sclerosis (N=12) | **0.37** | *0.04* | *3.15* | 1 | **1.35** | *0.36* | *5.08* | 4 | **0.32** | *0.04* | *2.73* | 1 |
| Cumulative Exposure to **Vinyl Chloride.** Reference group has no exposure to vinyl chloride | | | | | | | | | | | | |
| **Underlying**  **Cause of Death** | Low Exposure  **HR** LCL UCL N | | | | Medium Exposure  **HR** LCL UCL N | | | | High Exposure  **HR** LCL UCL N | | | |
| ALS (N=21) | **1.22** | *0.33* | *4.51* | 4 | **0.91** | *0.22* | *3.87* | 3 | **2.21** | *0.71* | *6.86* | 8 |
| Kidney Disease (N=37) | **1.69** | *0.72* | *3.96* | 11 | **1.18** | *0.47* | *3.01* | 8 | **1.03** | *0.39* | *2.72* | 7 |
| Liver Disease (N=191) | **0.90** | *0.59* | *1.37* | 34 | **1.16** | *0.79* | *1.72* | 45 | **1.12** | *0.76* | *1.67* | 44 |
| **Smoking-related Diseases** (not known to be related to solvent exposure) | | | | | | | | | | | | |
| **Underlying**  **Cause of Death** | Low Exposure  **HR** LCL UCL N | | | | Medium Exposure  **HR** LCL UCL N | | | | High Exposure  **HR** LCL UCL N | | | |
| Stomach Cancer (N=35) | **1.01** | *0.37* | *2.74* | 6 | **0.99** | *0.36* | *2.72* | 6 | **1.31** | *0.53* | *3.24* | 9 |
| COPD (N=47) | **1.26** | *0.60* | *2.63* | 13 | **0.72** | *0.31* | *1.71* | 8 | **0.44** | *0.16* | *1.20* | 5 |
| Cardiovascular (N=1,391) | **1.05** | *0.90* | *1.22* | 270 | **1.08** | *0.93* | *1.25* | 289 | **1.08** | *0.93* | *1.25* | 308 |

| **Cumulative Exposure to Trichloroethylene (TCE). Reference group has no exposure to TCE.** | | | | | | | | | | | | |
| --- | --- | --- | --- | --- | --- | --- | --- | --- | --- | --- | --- | --- |
| **Underlying**  **Cause of Death** | Low Exposure  **HR** LCL UCL N | | | | Medium Exposure  **HR** LCL UCL N | | | | High Exposure  **HR** LCL UCL N | | | |
| All cancers (N=1,078) | **1.02** | *0.86* | *1.21* | 219 | **1.05** | *0.89* | *1.26* | 210 | **1.00** | *0.84* | *1.19* | 211 |
| **Diseases of Primary Interest** | | | | | | | | | | | | |
| **Underlying**  **Cause of Death** | Low Exposure  **HR** LCL UCL N | | | | Medium Exposure  **HR** LCL UCL N | | | | High Exposure  **HR** LCL UCL N | | | |
| Kidney Cancer (N=42) | **1.54** | *0.65* | *3.61* | 11 | **1.21** | *0.47* | *3.09* | 8 | **1.52** | *0.64* | *3.61* | 11 |
| Bladder Cancer (N=11) | **0.00** | *0.00* |  | 0 | **2.69** | *0.63* | *11.46* | 5 | **0.92** | *0.15* | *5.55* | 2 |
| Liver Cancer (N=51) | **1.02** | *0.48* | *2.15* | 12 | **1.04** | *0.47* | *2.27* | 11 | **0.86** | *0.37* | *1.97* | 9 |
| Esophageal Cancer (N=35) | **0.97** | *0.42* | *2.27* | 9 | **0.43** | *0.14* | *1.32* | 4 | **0.73** | *0.29* | *1.83* | 7 |
| Hematopoietic Cancers (N=165) | **1.48** | *0.96* | *2.28* | 37 | **1.16** | *0.72* | *1.88* | 27 | **1.27** | *0.80* | *2.03* | 31 |
| Multiple Myeloma (N=17) | **2.09** | *0.66* | *6.62* | 7 | **1.29** | *0.34* | *4.88* | 4 | **0.00** | *0.00* |  | 0 |
| Leukemia (N=66) | **2.00** | *1.00* | *4.00* | 16 | **1.54** | *0.71* | *3.36* | 11 | **1.81** | *0.85* | *3.85* | 13 |
| Non-Hodgkin Lymphoma (N=58) | **0.90** | *0.42* | *1.92* | 10 | **0.75** | *0.33* | *1.70* | 8 | **1.15** | *0.56* | *2.34* | 13 |
| Hodgkin (N=24) | **1.52** | *0.42* | *5.59* | 4 | **1.63** | *0.43* | *6.12* | 4 | **1.97** | *0.55* | *7.03* | 5 |
| **Diseases of Secondary Interest** | | | | | | | | | | | | |
| **Underlying**  **Cause of Death** | Low Exposure  **HR** LCL UCL N | | | | Medium Exposure  **HR** LCL UCL N | | | | High Exposure  **HR** LCL UCL N | | | |
| Pancreatic Cancer (N=57) | **0.57** | *0.27* | *1.19* | 10 | **0.62** | *0.30* | *1.30* | 10 | **0.56** | *0.27* | *1.16* | 10 |
| Colorectal Cancers (N=110) | **0.81** | *0.47* | *1.39* | 20 | **0.84** | *0.47* | *1.48* | 18 | **1.05** | *0.62* | *1.77* | 24 |
| Rectal Cancer (N=24) | **1.23** | *0.44* | *3.41* | 7 | **0.58** | *0.15* | *2.24* | 3 | **1.13** | *0.38* | *3.32* | 6 |
| Colon Cancer (N=86) | **0.68** | *0.36* | *1.31* | 13 | **0.91** | *0.48* | *1.72* | 15 | **1.02** | *0.56* | *1.87* | 18 |
| Lung Cancer (N=237) | **1.20** | *0.84* | *1.72* | 55 | **1.25** | *0.87* | *1.79* | 55 | **1.16** | *0.80* | *1.68* | 53 |
| Brain Cancer (N=74) | **1.05** | *0.54* | *2.03* | 14 | **1.19** | *0.62* | *2.29* | 15 | **0.93** | *0.47* | *1.85* | 13 |
| Soft-tissue Cancers (N=29) | **0.50** | *0.14* | *1.83* | 3 | **1.37** | *0.51* | *3.68* | 7 | **0.37** | *0.08* | *1.71* | 2 |
| Oral cancers (N=26) | **2.05** | *0.74* | *5.69* | 9 | **0.24** | *0.03* | *2.03* | 1 | **1.68** | *0.57* | *5.01* | 7 |
| Prostate Cancer (N=18) | **1.27** | *0.41* | *3.98* | 6 | **0.42** | *0.09* | *2.11* | 2 | **0.60** | *0.15* | *2.41* | 3 |
| Breast Cancer (female) (N=10) | **2.08** | *0.40* | *10.76* | 2 | **0.00** | *0.00* |  | 0 | **0.00** | *0.00* |  | 0 |
| Multiple Sclerosis (N=12) | **0.35** | *0.04* | *2.98* | 1 | **1.34** | *0.36* | *5.08* | 4 | **0.32** | *0.04* | *2.73* | 1 |
| Cumulative Exposure to **Trichloroethylene** (TCE). Reference group has no exposure to TCE. | | | | | | | | | | | | |
| **Underlying**  **Cause of Death** | Low Exposure  **HR** LCL UCL N | | | | Medium Exposure  **HR** LCL UCL N | | | | High Exposure  **HR** LCL UCL N | | | |
| ALS (N=21) | **0.91** | *0.25* | *3.23* | 4 | **0.87** | *0.21* | *3.57* | 3 | **1.93** | *0.65* | *5.79* | 8 |
| Kidney Disease (N=37) | **1.63** | *0.69* | *3.82* | 11 | **1.18** | *0.46* | *2.99* | 8 | **1.01** | *0.38* | *2.67* | 7 |
| Liver Disease (N=191) | **0.83** | *0.54* | *1.29* | 31 | **1.35** | *0.92* | *1.98* | 50 | **1.17** | *0.79* | *1.74* | 45 |
| **Smoking-related Diseases** (not known to be related to solvent exposure) | | | | | | | | | | | | |
| **Underlying**  **Cause of Death** | Low Exposure  **HR** LCL UCL N | | | | Medium Exposure  **HR** LCL UCL N | | | | High Exposure  **HR** LCL UCL N | | | |
| Stomach Cancer (N=35) | **1.43** | *0.58* | *3.53* | 9 | **0.93** | *0.31* | *2.75* | 5 | **1.46** | *0.58* | *3.67* | 9 |
| COPD (N=47) | **1.13** | *0.53* | *2.40* | 12 | **0.90** | *0.40* | *2.00* | 10 | **0.34** | *0.11* | *1.02* | 4 |
| Cardiovascular (N=1,391) | **1.05** | *0.90* | *1.22* | 286 | **1.06** | *0.91* | *1.23* | 279 | **1.11** | *0.96* | *1.29* | 317 |

| **Cumulative Exposure to Tetrachloroethylene (PCE). Reference group has no exposure to PCE.** | | | | | | | | | | | | |
| --- | --- | --- | --- | --- | --- | --- | --- | --- | --- | --- | --- | --- |
| **Underlying**  **Cause of Death** | Low Exposure  **HR** LCL UCL N | | | | Medium Exposure  **HR** LCL UCL N | | | | High Exposure  **HR** LCL UCL N | | | |
| All cancers (N=1,078) | **1.10** | *0.92* | *1.30* | 210 | **1.08** | *0.91* | *1.29* | 211 | **0.96** | *0.81* | *1.15* | 209 |
| **Diseases of Primary Interest** | | | | | | | | | | | | |
| **Underlying**  **Cause of Death** | Low Exposure  **HR** LCL UCL N | | | | Medium Exposure  **HR** LCL UCL N | | | | High Exposure  **HR** LCL UCL N | | | |
| Kidney Cancer (N=42) | **1.40** | *0.54* | *3.58* | 8 | **1.82** | *0.75* | *4.42* | 11 | **1.59** | *0.66* | *3.86* | 11 |
| Bladder Cancer (N=11) | **0.55** | *0.06* | *5.35* | 1 | **1.62** | *0.32* | *8.10* | 3 | **1.24** | *0.25* | *6.21* | 3 |
| Liver Cancer (N=51) | **1.17** | *0.55* | *2.49* | 12 | **0.96** | *0.43* | *2.14* | 10 | **0.82** | *0.36* | *1.89* | 9 |
| Esophageal Cancer (N=35) | **1.27** | *0.57* | *2.81* | 11 | **0.55** | *0.20* | *1.55* | 5 | **0.41** | *0.13* | *1.26* | 4 |
| Hematopoietic Cancers (N=165) | **1.62** | *1.05* | *2.48* | 38 | **1.08** | *0.66* | *1.77* | 25 | **1.18** | *0.74* | *1.89* | 30 |
| Multiple Myeloma (N=17) | **2.24** | *0.67* | *7.51* | 6 | **1.06** | *0.24* | *4.59* | 3 | **0.58** | *0.11* | *3.11* | 2 |
| Leukemia (N=66) | **1.95** | *0.99* | *3.85* | 16 | **1.42** | *0.66* | *3.07* | 11 | **1.36** | *0.62* | *2.95* | 11 |
| Non-Hodgkin Lymphoma (N=58) | **1.18** | *0.58* | *2.42* | 12 | **0.58** | *0.23* | *1.46* | 6 | **1.14** | *0.56* | *2.32* | 13 |
| Hodgkin (N=24) | **1.57** | *0.43* | *5.74* | 4 | **2.10** | *0.60* | *7.34* | 5 | **1.55** | *0.40* | *5.92* | 4 |
| **Diseases of Secondary Interest** | | | | | | | | | | | | |
| **Underlying**  **Cause of Death** | Low Exposure  **HR** LCL UCL N | | | | Medium Exposure  **HR** LCL UCL N | | | | High Exposure  **HR** LCL UCL N | | | |
| Pancreatic Cancer (N=57) | **0.67** | *0.32* | *1.40* | 10 | **0.58** | *0.27* | *1.26* | 9 | **0.61** | *0.30* | *1.26* | 11 |
| Colorectal Cancers (N=110) | **0.86** | *0.49* | *1.50* | 18 | **1.08** | *0.64* | *1.84* | 23 | **0.79** | *0.45* | *1.39* | 19 |
| Rectal Cancer (N=24) | **1.19** | *0.41* | *3.45* | 6 | **0.96** | *0.31* | *2.97* | 5 | **0.70** | *0.21* | *2.35* | 4 |
| Colon Cancer (N=86) | **0.75** | *0.38* | *1.47* | 12 | **1.13** | *0.62* | *2.05* | 18 | **0.82** | *0.43* | *1.55* | 15 |
| Lung Cancer (N=237) | **1.33** | *0.93* | *1.90* | 56 | **1.27** | *0.88* | *1.83* | 55 | **1.08** | *0.75* | *1.57* | 51 |
| Brain Cancer (N=74) | **1.16** | *0.60* | *2.25* | 14 | **1.08** | *0.54* | *2.14* | 13 | **1.08** | *0.56* | *2.08* | 15 |
| Soft-tissue Cancers (N=29) | **0.79** | *0.25* | *2.52* | 4 | **1.41** | *0.52* | *3.80* | 7 | **0.18** | *0.02* | *1.41* | 1 |
| Oral cancers (N=26) | **1.89** | *0.63* | *5.66* | 7 | **0.80** | *0.20* | *3.24* | 3 | **1.80** | *0.59* | *5.46* | 7 |
| Prostate Cancer (N=18) | **0.70** | *0.18* | *2.83* | 3 | **0.43** | *0.09* | *2.16* | 2 | **1.16** | *0.37* | *3.63* | 6 |
| Breast Cancer (female) (N=10) | **1.95** | *0.38* | *10.13* | 2 | **0.00** | *0.00* |  | 0 | **0.00** | *0.00* |  | 0 |
| Multiple Sclerosis (N=12) | **0.00** | *0.00* |  | 0 | **1.04** | *0.24* | *4.39* | 3 | **0.95** | *0.22* | *4.09* | 3 |
| Cumulative Exposure to **Tetrachloroethylene** (PCE). Reference group has no exposure to PCE. | | | | | | | | | | | | |
| **Underlying**  **Cause of Death** | Low Exposure  **HR** LCL UCL N | | | | Medium Exposure  **HR** LCL UCL N | | | | High Exposure  **HR** LCL UCL N | | | |
| ALS (N=21) | **0.69** | *0.13* | *3.55* | 2 | **1.58** | *0.45* | *5.50* | 5 | **1.96** | *0.64* | *6.02* | 8 |
| Kidney Disease (N=37) | **1.58** | *0.66* | *3.78* | 10 | **1.37** | *0.55* | *3.40* | 9 | **0.98** | *0.37* | *2.60* | 7 |
| Liver Disease (N=191) | **0.87** | *0.57* | *1.34* | 32 | **1.27** | *0.86* | *1.86* | 48 | **1.06** | *0.71* | *1.57* | 43 |
| **Smoking-related Diseases** (not known to be related to solvent exposure) | | | | | | | | | | | | |
| **Underlying**  **Cause of Death** | Low Exposure  **HR** LCL UCL N | | | | Medium Exposure  **HR** LCL UCL N | | | | High Exposure  **HR** LCL UCL N | | | |
| Stomach Cancer (N=35) | **0.70** | *0.22* | *2.20* | 4 | **1.02** | *0.37* | *2.80* | 6 | **1.56** | *0.66* | *3.69* | 11 |
| COPD (N=47) | **1.30** | *0.62* | *2.72* | 13 | **0.74** | *0.31* | *1.74* | 8 | **0.42** | *0.15* | *1.15* | 5 |
| Cardiovascular (N=1,391) | **1.02** | *0.88* | *1.20* | 259 | **1.02** | *0.88* | *1.19* | 270 | **1.12** | *0.97* | *1.29* | 334 |

**Notes on the tables**

Hazard ratios were obtained using the Cox extended model with categorical cumulative exposure as a time-varying variable and age as the time variable. The hazard ratios were adjusted by sex, race, rank, and education level. The categorical cumulative exposure variable was lagged 10 years.

The analyses were internal to the Camp Lejeune cohort.

Because of sparse data, regressions were not conducted for the following diseases: laryngeal cancer, male breast cancer, cervical cancer, aplastic anemia, and Parkinson’s disease.

Liver cancer includes cancers of the biliary passages, liver and gall bladder.

Lung cancer includes cancers of the trachea, bronchus, and lung.

Oral cancers include cancers of the buccal cavity and pharynx.

Cardiovascular disease includes diseases of the heart and other diseases of the circulatory system.
