# Supplementary material for: Evaluation of mortality among marines and navy personnel exposed to contaminated drinking water at USMC base Camp Lejeune: a retrospective cohort study
Source: Environ Health. 2014 Feb 19;13:10. doi: 10.1186/1476-069X-13-10 (PMC3943370; doi:10.1186/1476-069X-13-10)
Supplement: Additional file 3: Table S2 — Cumulative Exposures and Underlying Cause of Death. [file 1476-069X-13-10-S3.docx]

**Additional file 3: Table S2: Cumulative Exposures and Underlying Cause of Death**

(10 year exposure lag, adjusted). Camp Lejeune cohort (N = 154,932).

| Underlying Cause | Cumulative Exposure | | | Beta Coefficient | | Standard Error | | P-value | | Lower 95% CL | | Upper 95% CL |
| --- | --- | --- | --- | --- | --- | --- | --- | --- | --- | --- | --- | --- |
| All Cancers (N=1,078) | PCE | | | -0.00005 | | 0.00007 | | 0.47 | | -0.00019 | | 0.00009 |
| All Cancers | TCE | | | 0.00000 | | 0.00001 | | 0.76 | | -0.00002 | | 0.00002 |
| All Cancers | VC | | | -0.00008 | | 0.00009 | | 0.40 | | -0.00026 | | 0.00010 |
| All Cancers | Benzene | | | -0.00045 | | 0.00043 | | 0.30 | | -0.00129 | | 0.00039 |
| All Cancers | TVOC | | | 0.00000 | | 0.00000 | | 0.65 | | -0.00001 | | 0.00001 |
| **Diseases of Primary Interest** | | | | | | | | | | | | |
| Underlying Cause | | Cumulative Exposure | Beta Coefficient | | Standard Error | | P-value | | Lower 95% CL | | Upper 95% CL | |
| Kidney Cancer (N=42) | | PCE | 0.00009 | | 0.00029 | | 0.76 | | -0.00048 | | 0.00066 | |
| Kidney Cancer | | TCE | 0.00002 | | 0.00003 | | 0.57 | | -0.00004 | | 0.00008 | |
| Kidney Cancer | | VC | 0.00021 | | 0.00043 | | 0.63 | | -0.00063 | | 0.00105 | |
| Kidney Cancer | | Benzene | 0.00078 | | 0.00194 | | 0.69 | | -0.00303 | | 0.00459 | |
| Kidney Cancer | | TVOC | 0.00001 | | 0.00002 | | 0.59 | | -0.00003 | | 0.00005 | |
|  | |  |  | |  | |  | |  | |  | |
| Bladder Cancer (N=11) | | PCE | 0.00010 | | 0.00047 | | 0.83 | | -0.00082 | | 0.00102 | |
| Bladder Cancer | | TCE | 0.00002 | | 0.00006 | | 0.77 | | -0.00010 | | 0.00014 | |
| Bladder Cancer | | VC | 0.00028 | | 0.00081 | | 0.73 | | -0.00131 | | 0.00187 | |
| Bladder Cancer | | Benzene | 0.00211 | | 0.00348 | | 0.54 | | -0.00473 | | 0.00895 | |
| Bladder Cancer | | TVOC | 0.00001 | | 0.00004 | | 0.76 | | -0.00007 | | 0.00009 | |
|  | |  |  | |  | |  | |  | |  | |
| Liver Cancer* (N=51) | | PCE | -0.00024 | | 0.00039 | | 0.53 | | -0.00101 | | 0.00053 | |
| Liver Cancer | | TCE | 0.00001 | | 0.00003 | | 0.65 | | -0.00005 | | 0.00007 | |
| Liver Cancer | | VC | 0.00008 | | 0.00043 | | 0.85 | | -0.00076 | | 0.00092 | |
| Liver Cancer | | Benzene | 0.00022 | | 0.00197 | | 0.91 | | -0.00365 | | 0.00409 | |
| Liver Cancer | | TVOC | 0.00001 | | 0.00002 | | 0.72 | | -0.00003 | | 0.00005 | |
|  | |  |  | |  | |  | |  | |  | |
| Esophageal Cancer (N=35) | | PCE | -0.00169 | | 0.00090 | | 0.06 | | -0.00346 | | 0.00008 | |
| Esophageal Cancer | | TCE | -0.00006 | | 0.00004 | | 0.15 | | -0.00014 | | 0.00002 | |
| Esophageal Cancer | | VC | -0.00106 | | 0.00066 | | 0.11 | | -0.00236 | | 0.00024 | |
| Esophageal Cancer | | Benzene | -0.00288 | | 0.00270 | | 0.29 | | -0.00819 | | 0.00243 | |
| Esophageal Cancer | | TVOC | -0.00004 | | 0.00003 | | 0.12 | | -0.00010 | | 0.00002 | |
|  | |  |  | |  | |  | |  | |  | |
| Hematopoietic Cancers (N=165) | | PCE | 0.00005 | | 0.00019 | | 0.79 | | -0.00032 | | 0.00042 | |
| Hematopoietic Cancers | | TCE | 0.00000 | | 0.00002 | | 0.85 | | -0.00004 | | 0.00004 | |
| Hematopoietic Cancers | | VC | 0.00001 | | 0.00024 | | 0.96 | | -0.00046 | | 0.00048 | |
| Hematopoietic Cancers | | Benzene | -0.00017 | | 0.00112 | | 0.88 | | -0.00237 | | 0.00203 | |
| Hematopoietic Cancers | | TVOC | 0.00000 | | 0.00001 | | 0.86 | | -0.00002 | | 0.00002 | |
| Underlying Cause | | Cumulative Exposure | Beta Coefficient | | Standard Error | | P-value | | Lower 95% CL | | Upper 95% CL | |
| Hodgkin Lymphoma (N=24) | | PCE | 0.00001 | | 0.00055 | | 0.98 | | -0.00107 | | 0.00109 | |
| Hodgkin Lymphoma | | TCE | 0.00005 | | 0.00004 | | 0.20 | | -0.00003 | | 0.00013 | |
| Hodgkin Lymphoma | | VC | 0.00056 | | 0.00059 | | 0.34 | | -0.00060 | | 0.00172 | |
| Hodgkin Lymphoma | | Benzene | 0.00203 | | 0.00276 | | 0.46 | | -0.00339 | | 0.00745 | |
| Hodgkin Lymphoma | | TVOC | 0.00003 | | 0.00003 | | 0.24 | | -0.00003 | | 0.00009 | |
|  | |  |  | |  | |  | |  | |  | |
| Non-Hodgkin Lymphoma (N=58) | | PCE | 0.00004 | | 0.00031 | | 0.89 | | -0.00057 | | 0.00065 | |
| Non-Hodgkin Lymphoma | | TCE | 0.00000 | | 0.00003 | | 0.99 | | -0.00006 | | 0.00006 | |
| Non-Hodgkin Lymphoma | | VC | -0.00010 | | 0.00041 | | 0.81 | | -0.00091 | | 0.00071 | |
| Non-Hodgkin Lymphoma | | Benzene | -0.00097 | | 0.00191 | | 0.61 | | -0.00472 | | 0.00278 | |
| Non-Hodgkin Lymphoma | | TVOC | 0.00000 | | 0.00002 | | 0.99 | | -0.00004 | | 0.00004 | |
|  | |  |  | |  | |  | |  | |  | |
| Leukemias (N=66) | | PCE | 0.00006 | | 0.00031 | | 0.86 | | -0.00055 | | 0.00067 | |
| Leukemias | | TCE | 0.00002 | | 0.00003 | | 0.46 | | -0.00004 | | 0.00008 | |
| Leukemias | | VC | 0.00034 | | 0.00037 | | 0.35 | | -0.00039 | | 0.00107 | |
| Leukemias | | Benzene | 0.00168 | | 0.00166 | | 0.31 | | -0.00158 | | 0.00494 | |
| Leukemias | | TVOC | 0.00001 | | 0.00002 | | 0.44 | | -0.00003 | | 0.00005 | |
|  | |  |  | |  | |  | |  | |  | |
| Multiple Myeloma (N=17) | | PCE | 0.00008 | | 0.00047 | | 0.86 | | -0.00084 | | 0.00100 | |
| Multiple Myeloma | | TCE | -0.00017 | | 0.00009 | | 0.05 | | -0.00035 | | 0.00001 | |
| Multiple Myeloma | | VC | -0.00257 | | 0.00134 | | 0.06 | | -0.00520 | | 0.00006 | |
| Multiple Myeloma | | Benzene | -0.01174 | | 0.00608 | | 0.05 | | -0.02369 | | 0.00021 | |
| Multiple Myeloma | | TVOC | -0.00010 | | 0.00005 | | 0.06 | | -0.00020 | | 0.00000 | |
| **Diseases of Secondary Interest** | | | | | | | | | | | | |
| Underlying Cause | | Cumulative Exposure | Beta Coefficient | | Standard Error | | P-value | | Lower 95% CL | | Upper 95% CL | |
| Pancreatic Cancer (N=57) | | PCE | -0.00011 | | 0.00031 | | 0.73 | | -0.00072 | | 0.00050 | |
| Pancreatic Cancer | | TCE | -0.00003 | | 0.00003 | | 0.25 | | -0.00009 | | 0.00003 | |
| Pancreatic Cancer | | VC | -0.00053 | | 0.00044 | | 0.22 | | -0.00139 | | 0.00033 | |
| Pancreatic Cancer | | Benzene | -0.00194 | | 0.00192 | | 0.31 | | -0.00571 | | 0.00183 | |
| Pancreatic Cancer | | TVOC | -0.00002 | | 0.00002 | | 0.24 | | -0.00006 | | 0.00002 | |
|  | |  |  | |  | |  | |  | |  | |
| Colorectal Cancer (N=110) | | PCE | -0.00029 | | 0.00028 | | 0.31 | | -0.00084 | | 0.00026 | |
| Colorectal Cancer | | TCE | 0.00000 | | 0.00002 | | 0.85 | | -0.00004 | | 0.00004 | |
| Colorectal Cancer | | VC | 0.00002 | | 0.00029 | | 0.95 | | -0.00055 | | 0.00059 | |
| Colorectal Cancer | | Benzene | -0.00007 | | 0.00133 | | 0.96 | | -0.00268 | | 0.00254 | |
| Colorectal Cancer | | TVOC | 0.00000 | | 0.00001 | | 0.93 | | -0.00002 | | 0.00002 | |
|  | |  |  | |  | |  | |  | |  | |
| Colon Cancer (N=86) | | PCE | -0.00019 | | 0.00029 | | 0.53 | | -0.00076 | | 0.00038 | |
| Colon Cancer | | TCE | 0.00001 | | 0.00002 | | 0.72 | | -0.00003 | | 0.00005 | |
| Colon Cancer | | VC | 0.00011 | | 0.00033 | | 0.74 | | -0.00054 | | 0.00076 | |
| Underlying Cause | | Cumulative Exposure | Beta Coefficient | | Standard Error | | P-value | | Lower 95% CL | | Upper 95% CL | |
| Colon Cancer | | Benzene | 0.00038 | | 0.00149 | | 0.80 | | -0.00255 | | 0.00331 | |
| Colon Cancer | | TVOC | 0.00000 | | 0.00001 | | 0.77 | | -0.00002 | | 0.00002 | |
|  | |  |  | |  | |  | |  | |  | |
| Rectal Cancer (N=24) | | PCE | -0.00079 | | 0.00080 | | 0.32 | | -0.00236 | | 0.00078 | |
| Rectal Cancer | | TCE | -0.00001 | | 0.00004 | | 0.79 | | -0.00009 | | 0.00007 | |
| Rectal Cancer | | VC | -0.00029 | | 0.00065 | | 0.65 | | -0.00157 | | 0.00099 | |
| Rectal Cancer | | Benzene | -0.00161 | | 0.00298 | | 0.59 | | -0.00747 | | 0.00425 | |
| Rectal Cancer | | TVOC | -0.00001 | | 0.00003 | | 0.74 | | -0.00007 | | 0.00005 | |
|  | |  |  | |  | |  | |  | |  | |
| Lung Cancer** (N=237) | | PCE | -0.00021 | | 0.00017 | | 0.21 | | -0.00054 | | 0.00012 | |
| Lung Cancer | | TCE | 0.00001 | | 0.00001 | | 0.50 | | -0.00001 | | 0.00003 | |
| Lung Cancer | | VC | 0.00005 | | 0.00019 | | 0.78 | | -0.00032 | | 0.00042 | |
| Lung Cancer | | Benzene | 0.00017 | | 0.00088 | | 0.85 | | -0.00156 | | 0.00190 | |
| Lung Cancer | | TVOC | 0.00000 | | 0.00001 | | 0.61 | | -0.00002 | | 0.00002 | |
|  | |  |  | |  | |  | |  | |  | |
| Soft Tissue Cancers (N=29) | | PCE | -0.00113 | | 0.00097 | | 0.24 | | -0.00304 | | 0.00078 | |
| Soft Tissue Cancers | | TCE | -0.00003 | | 0.00005 | | 0.56 | | -0.00013 | | 0.00007 | |
| Soft Tissue Cancers | | VC | -0.00055 | | 0.00073 | | 0.45 | | -0.00198 | | 0.00088 | |
| Soft Tissue Cancers | | Benzene | -0.00255 | | 0.00333 | | 0.44 | | -0.00909 | | 0.00399 | |
| Soft Tissue Cancers | | TVOC | -0.00002 | | 0.00003 | | 0.51 | | -0.00008 | | 0.00004 | |
|  | |  |  | |  | |  | |  | |  | |
| Brain Cancer (N=74) | | PCE | 0.00031 | | 0.00019 | | 0.10 | | -0.00006 | | 0.00068 | |
| Brain Cancer | | TCE | -0.00001 | | 0.00003 | | 0.68 | | -0.00007 | | 0.00005 | |
| Brain Cancer | | VC | -0.00010 | | 0.00036 | | 0.77 | | -0.00081 | | 0.00061 | |
| Brain Cancer | | Benzene | -0.00066 | | 0.00164 | | 0.69 | | -0.00388 | | 0.00256 | |
| Brain Cancer | | TVOC | 0.00000 | | 0.00002 | | 0.75 | | -0.00004 | | 0.00004 | |
|  | |  |  | |  | |  | |  | |  | |
| Oral Cancer*** (N=26) | | PCE | 0.00042 | | 0.00023 | | 0.06 | | -0.00003 | | 0.00087 | |
| Oral Cancer | | TCE | 0.00000 | | 0.00004 | | 0.92 | | -0.00008 | | 0.00008 | |
| Oral Cancer | | VC | -0.00004 | | 0.00063 | | 0.94 | | -0.00128 | | 0.00120 | |
| Oral Cancer | | Benzene | -0.00168 | | 0.00314 | | 0.59 | | -0.00785 | | 0.00449 | |
| Oral Cancer | | TVOC | 0.00000 | | 0.00003 | | 0.99 | | -0.00006 | | 0.00006 | |
|  | |  |  | |  | |  | |  | |  | |
| Prostate Cancer (N=18) | | PCE | 0.00035 | | 0.00025 | | 0.17 | | -0.00014 | | 0.00084 | |
| Prostate Cancer | | TCE | -0.00008 | | 0.00006 | | 0.20 | | -0.00020 | | 0.00004 | |
| Prostate Cancer | | VC | -0.00075 | | 0.00084 | | 0.37 | | -0.00241 | | 0.00090 | |
| Prostate Cancer | | Benzene | -0.00541 | | 0.00429 | | 0.21 | | -0.01383 | | 0.00301 | |
| Prostate Cancer | | TVOC | -0.00004 | | 0.00004 | | 0.27 | | -0.00011 | | 0.00003 | |
|  | |  |  | |  | |  | |  | |  | |
| Breast Cancer (females) (N=10) | | PCE | -0.00210 | | 0.00690 | | 0.76 | | -0.01566 | | 0.01146 | |
| Breast Cancer (females) | | TCE | 0.00000 | | 0.00330 | | 0.94 | | -0.00648 | | 0.00648 | |
| Breast Cancer (females) | | VC | -0.00137 | | 0.00700 | | 0.85 | | -0.01513 | | 0.01239 | |
| Breast Cancer (females) | | Benzene | -0.00220 | | 0.03210 | | 0.95 | | -0.06528 | | 0.06088 | |
| Underlying Cause | | Cumulative Exposure | Beta Coefficient | | Standard Error | | P-value | | Lower 95% CL | | Upper 95% CL | |
| Breast Cancer (females) | | TVOC | 0.00000 | | 0.00020 | | 0.89 | | -0.00039 | | 0.00039 | |
|  | |  |  | |  | |  | |  | |  | |
| Liver Diseases (N=191 | | PCE | -0.00012 | | 0.00018 | | 0.52 | | -0.00047 | | 0.00023 | |
| Liver Diseases | | TCE | 0.00001 | | 0.00001 | | 0.39 | | -0.00001 | | 0.00003 | |
| Liver Diseases | | VC | 0.00014 | | 0.00021 | | 0.48 | | -0.00027 | | 0.00055 | |
| Liver Diseases | | Benzene | 0.00055 | | 0.00094 | | 0.56 | | -0.00130 | | 0.00240 | |
| Liver Diseases | | TVOC | 0.00001 | | 0.00001 | | 0.43 | | -0.00001 | | 0.00003 | |
|  | |  |  | |  | |  | |  | |  | |
| Kidney Diseases (N=37) | | PCE | 0.00018 | | 0.00027 | | 0.51 | | -0.00035 | | 0.00071 | |
| Kidney Diseases | | TCE | -0.00001 | | 0.00003 | | 0.86 | | -0.00007 | | 0.00005 | |
| Kidney Diseases | | VC | 0.00002 | | 0.00048 | | 0.97 | | -0.00092 | | 0.00096 | |
| Kidney Diseases | | Benzene | -0.00057 | | 0.00224 | | 0.80 | | -0.00497 | | 0.00383 | |
| Kidney Diseases | | TVOC | 0.00000 | | 0.00002 | | 0.93 | | -0.00004 | | 0.00004 | |
|  | |  |  | |  | |  | |  | |  | |
| Multiple Sclerosis (N=12) | | PCE | -0.0556 | | 0.1146 | | 0.63 | | -.28079 | | .16959 | |
| Multiple Sclerosis | | TCE | -0.0727 | | 0.0926 | | 0.43 | | -.25466 | | .10926 | |
| Multiple Sclerosis | | VC | -0.0777 | | 0.1118 | | 0.49 | | -.29739 | | .14199 | |
| Multiple Sclerosis | | Benzene | -0.1652 | | 0.1265 | | 0.19 | | -.41377 | | .08337 | |
| Multiple Sclerosis | | TVOC | -0.0789 | | 0.0908 | | 0.39 | | -.25732 | | .09952 | |
|  | |  |  | |  | |  | |  | |  | |
| Amyotrophic Lateral Sclerosis (N=21) | | PCE | 0.00039 | | 0.00021 | | 0.06 | | -0.00002 | | 0.00080 | |
| ALS | | TCE | 0.00007 | | 0.00003 | | 0.04 | | 0.00001 | | 0.00013 | |
| ALS | | VC | 0.00110 | | 0.00046 | | 0.02 | | 0.00020 | | 0.00200 | |
| ALS | | Benzene | 0.00464 | | 0.00208 | | 0.03 | | 0.00055 | | 0.00873 | |
| ALS | | TVOC | 0.00005 | | 0.00002 | | 0.03 | | 0.00001 | | 0.00009 | |
| **Smoking-related Diseases** (not known to be related to solvent exposure) | | | | | | | | | | | | |
| Underlying Cause | | Cumulative Exposure | Beta Coefficient | | Standard Error | | P-value | | Lower 95% CL | | Upper 95% CL | |
| Stomach Cancer (N=35) | | PCE | 0.00018 | | 0.00029 | | 0.53 | | -0.00039 | | 0.00075 | |
| Stomach Cancer | | TCE | 0.00002 | | 0.00003 | | 0.53 | | -0.00004 | | 0.00008 | |
| Stomach Cancer | | VC | 0.00031 | | 0.00045 | | 0.50 | | -0.00057 | | 0.00119 | |
| Stomach Cancer | | Benzene | 0.00166 | | 0.00202 | | 0.41 | | -0.00231 | | 0.00563 | |
| Stomach Cancer | | TVOC | 0.00001 | | 0.00002 | | 0.52 | | -0.00003 | | 0.00005 | |
|  | |  |  | |  | |  | |  | |  | |
| Cardiovascular Disease† (N=1,391) | | PCE | 0.00004 | | 0.00006 | | 0.52 | | -0.00008 | | 0.00016 | |
| Cardiovascular Disease | | TCE | 0.00001 | | 0.00001 | | 0.26 | | -0.00001 | | 0.00003 | |
| Cardiovascular Disease | | VC | 0.00005 | | 0.00008 | | 0.55 | | -0.00011 | | 0.00021 | |
| Cardiovascular Disease | | Benzene | 0.00009 | | 0.00035 | | 0.79 | | -0.00060 | | 0.00078 | |
| Cardiovascular Disease | | TVOC | 0.00000 | | 0.00000 | | 0.31 | | 0.00000 | | 0.00000 | |
|  | |  |  | |  | |  | |  | |  | |
|  | |  |  | |  | |  | |  | |  | |
| Underlying Cause | | Cumulative Exposure | Beta Coefficient | | Standard Error | | P-value | | Lower 95% CL | | Upper 95% CL | |
| Chronic Obstructive Pulmonary Disease (N=47) | | PCE | -0.00065 | | 0.00055 | | 0.24 | | -0.00173 | | 0.00043 | |
| COPD | | TCE | -0.00006 | | 0.00004 | | 0.10 | | -0.00014 | | 0.00002 | |
| COPD | | VC | -0.00093 | | 0.00056 | | 0.10 | | -0.00203 | | 0.00017 | |
| COPD | | Benzene | -0.00467 | | 0.00261 | | 0.07 | | -0.00980 | | 0.00046 | |
| COPD | | TVOC | -0.00004 | | 0.00002 | | 0.09 | | -0.00008 | | 0.00000 | |

* Biliary passages, liver and gall bladder

** Trachea, bronchus, and lung

*** Buccal cavity and Pharynx.

† Includes diseases of the heart and other diseases of the circulatory system

Notes on the table

Hazard ratios were obtained using the Cox extended model with cumulative exposure as a time-varying variable and age as the time variable. The hazard ratios were adjusted by sex, race, rank, and education level. The cumulative exposure variable was lagged 10 years.

The analyses were internal to the Camp Lejeune cohort.

Because of sparse data, regressions were not conducted for the following diseases: male breast cancer, laryngeal cancer, cervical cancer, aplastic anemia, and Parkinson’s disease.

**Log Base 10 Cumulative Exposures and Underlying Cause of Death**

(10 year exposure lag, adjusted). Camp Lejeune cohort (N = 154,932).

| Underlying Cause | Log Cum. Exposure | Beta Coefficient | Standard Error | P-value | Lower 95% CL | Upper 95% CL |
| --- | --- | --- | --- | --- | --- | --- |
| All Cancers | PCE | 0.0035 | 0.0127 | 0.78 | -0.0215 | 0.0285 |
| All Cancers | TCE | 0.0035 | 0.0104 | 0.74 | -0.0169 | 0.0239 |
| All Cancers | VC | 0.0032 | 0.0126 | 0.80 | -0.0216 | 0.0280 |
| All Cancers | Benzene | 0.0016 | 0.0142 | 0.91 | -0.0263 | 0.0295 |
| All Cancers | TVOC | 0.0021 | 0.0103 | 0.83 | -0.0181 | 0.0223 |
| **Diseases of Primary Interest** | | | | | | |
| Underlying Cause | Log Cum. Exposure | Beta Coefficient | Standard Error | P-value | Lower 95% CL | Upper 95% CL |
| Kidney Cancer (N=42) | PCE | 0.0813 | 0.0695 | 0.24 | -0.0553 | 0.2179 |
| Kidney Cancer | TCE | 0.0571 | 0.0561 | 0.31 | -0.0531 | 0.1673 |
| Kidney Cancer | VC | 0.0742 | 0.0682 | 0.28 | -0.0598 | 0.2082 |
| Kidney Cancer | Benzene | 0.0643 | 0.0751 | 0.39 | -0.0833 | 0.2119 |
| Kidney Cancer | TVOC | 0.0633 | 0.0567 | 0.26 | -0.0481 | 0.1747 |
|  |  |  |  |  |  |  |
| Bladder Cancer (N=11) | PCE | 0.0443 | 0.1294 | 0.73 | -0.2100 | 0.2986 |
| Bladder Cancer | TCE | 0.0390 | 0.1071 | 0.72 | -0.1715 | 0.2495 |
| Bladder Cancer | VC | 0.0443 | 0.1284 | 0.73 | -0.2080 | 0.2966 |
| Bladder Cancer | Benzene | 0.1740 | 0.1670 | 0.30 | -0.1542 | 0.5022 |
| Bladder Cancer | TVOC | 0.0753 | 0.1146 | 0.51 | -0.1499 | 0.3005 |
|  |  |  |  |  |  |  |
| Liver Cancer* (N=51) | PCE | -0.0175 | 0.0582 | 0.76 | -0.1319 | 0.0969 |
| Liver Cancer | TCE | -0.0019 | 0.0480 | 0.97 | -0.0962 | 0.0924 |
| Liver Cancer | VC | -0.0118 | 0.0578 | 0.84 | -0.1254 | 0.1018 |
| Liver Cancer | Benzene | 0.0138 | 0.0662 | 0.84 | -0.1163 | 0.1439 |
| Liver Cancer | TVOC | -0.0057 | 0.0471 | 0.90 | -0.0983 | 0.0869 |
|  |  |  |  |  |  |  |
| Esophageal Cancer (N=35) | PCE | -0.0758 | 0.0655 | 0.25 | -0.2045 | 0.0529 |
| Esophageal Cancer | TCE | -0.0517 | 0.0536 | 0.33 | -0.1570 | 0.0536 |
| Esophageal Cancer | VC | -0.0677 | 0.0647 | 0.30 | -0.1948 | 0.0594 |
| Esophageal Cancer | Benzene | -0.3590 | 0.0743 | 0.63 | -0.5050 | -0.2130 |
| Esophageal Cancer | TVOC | -0.0492 | 0.0525 | 0.35 | -0.1524 | 0.0540 |
|  |  |  |  |  |  |  |
| Hematopoietic Cancers (N=165) | PCE | 0.0462 | 0.0340 | 0.17 | -0.0206 | 0.1130 |
| Hematopoietic Cancers | TCE | 0.0373 | 0.0276 | 0.18 | -0.0169 | 0.0915 |
| Hematopoietic Cancers | VC | 0.0456 | 0.0334 | 0.17 | -0.0200 | 0.1112 |
| Hematopoietic Cancers | Benzene | 0.0414 | 0.0378 | 0.27 | -0.0329 | 0.1157 |
| Hematopoietic Cancers | TVOC | 0.0420 | 0.0278 | 0.13 | -0.0126 | 0.0966 |
|  |  |  |  |  |  |  |
| Underlying Cause | Log Cum. Exposure | Beta Coefficient | Standard Error | P-value | Lower 95% CL | Upper 95% CL |
| Hodgkin Lymphoma (N=24) | PCE | 0.1047 | 0.0986 | 0.29 | -0.0890 | 0.2984 |
| Hodgkin Lymphoma | TCE | 0.0940 | 0.0809 | 0.25 | -0.0650 | 0.2530 |
| Hodgkin Lymphoma | VC | 0.1101 | 0.0976 | 0.26 | -0.0817 | 0.3019 |
| Hodgkin Lymphoma | Benzene | 0.1074 | 0.1100 | 0.33 | -0.1088 | 0.3236 |
| Hodgkin Lymphoma | TVOC | 0.0752 | 0.0799 | 0.35 | -0.0818 | 0.2322 |
|  |  |  |  |  |  |  |
| Non-Hodgkin Lymphoma (N=58) | PCE | -0.0053 | 0.0542 | 0.92 | -0.1118 | 0.1012 |
| Non-Hodgkin Lymphoma | TCE | -0.0083 | 0.0440 | 0.85 | -0.0948 | 0.0782 |
| Non-Hodgkin Lymphoma | VC | -0.0066 | 0.0532 | 0.90 | -0.1111 | 0.0979 |
| Non-Hodgkin Lymphoma | Benzene | -0.0329 | 0.0599 | 0.58 | -0.1506 | 0.0848 |
| Non-Hodgkin Lymphoma | TVOC | -0.0135 | 0.0437 | 0.76 | -0.0994 | 0.0724 |
|  |  |  |  |  |  |  |
| Leukemias (N=66) | PCE | 0.0901 | 0.0556 | 0.11 | -0.0192 | 0.1994 |
| Leukemias | TCE | 0.0801 | 0.0455 | 0.08 | -0.0093 | 0.1695 |
| Leukemias | VC | 0.0938 | 0.0549 | 0.09 | -0.0141 | 0.2017 |
| Leukemias | Benzene | 0.1276 | 0.0639 | 0.05 | 0.0020 | 0.2532 |
| Leukemias | TVOC | 0.0950 | 0.0467 | 0.04 | 0.0032 | 0.1868 |
|  |  |  |  |  |  |  |
| Multiple Myeloma (N=17) | PCE | 0.0074 | 0.1012 | 0.94 | -0.1915 | 0.2063 |
| Multiple Myeloma | TCE | -0.0146 | 0.0811 | 0.86 | -0.1740 | 0.1448 |
| Multiple Myeloma | VC | -0.0114 | 0.0983 | 0.91 | -0.2046 | 0.1818 |
| Multiple Myeloma | Benzene | -0.0612 | 0.1081 | 0.57 | -0.2736 | 0.1512 |
| Multiple Myeloma | TVOC | 0.0180 | 0.0833 | 0.83 | -0.1457 | 0.1817 |
| **Diseases of Secondary Interest** | | | | | | |
| Underlying Cause | Log Cum. Exposure | Beta Coefficient | Standard Error | P-value | Lower 95% CL | Upper 95% CL |
| Pancreatic Cancer (N=57) | PCE | -0.0929 | 0.0511 | 0.07 | -0.1933 | 0.0075 |
| Pancreatic Cancer | TCE | -0.0825 | 0.0417 | 0.05 | -0.1644 | -0.0006 |
| Pancreatic Cancer | VC | -0.0964 | 0.0503 | 0.06 | -0.1952 | 0.0024 |
| Pancreatic Cancer | Benzene | -0.0842 | 0.0572 | 0.14 | -0.1966 | 0.0282 |
| Pancreatic Cancer | TVOC | -0.0699 | 0.0409 | 0.09 | -0.1503 | 0.0105 |
|  |  |  |  |  |  |  |
| Colorectal Cancer (N=110) | PCE | -0.0225 | 0.0397 | 0.57 | -0.1005 | 0.0555 |
| Colorectal Cancer | TCE | -0.0168 | 0.0324 | 0.61 | -0.0805 | 0.0469 |
| Colorectal Cancer | VC | -0.0204 | 0.0392 | 0.60 | -0.0974 | 0.0566 |
| Colorectal Cancer | Benzene | -0.0285 | 0.0442 | 0.52 | -0.1154 | 0.0584 |
| Colorectal Cancer | TVOC | -0.0200 | 0.0319 | 0.53 | -0.0827 | 0.0427 |
|  |  |  |  |  |  |  |
| Colon Cancer (N=86) | PCE | -0.0241 | 0.0454 | 0.60 | -0.1133 | 0.0651 |
| Colon Cancer | TCE | -0.0218 | 0.0370 | 0.56 | -0.0945 | 0.0509 |
| Colon Cancer | VC | -0.0236 | 0.0448 | 0.60 | -0.1116 | 0.0644 |
| Underlying Cause | Log Cum. Exposure | Beta Coefficient | Standard Error | P-value | Lower 95% CL | Upper 95% CL |
| Colon Cancer | Benzene | -0.0348 | 0.0505 | 0.49 | -0.1340 | 0.0644 |
| Colon Cancer | TVOC | -0.0215 | 0.0365 | 0.56 | -0.0932 | 0.0502 |
|  |  |  |  |  |  |  |
| Rectal Cancer (N=24) | PCE | -0.0182 | 0.0821 | 0.82 | -0.1795 | 0.1431 |
| Rectal Cancer | TCE | -0.0010 | 0.0678 | 0.99 | -0.1342 | 0.1322 |
| Rectal Cancer | VC | -0.0109 | 0.0814 | 0.89 | -0.1709 | 0.1491 |
| Rectal Cancer | Benzene | -0.0081 | 0.0925 | 0.93 | -0.1899 | 0.1737 |
| Rectal Cancer | TVOC | -0.0157 | 0.0659 | 0.81 | -0.1452 | 0.1138 |
|  |  |  |  |  |  |  |
| Lung Cancer** (N=237) | PCE | 0.0248 | 0.0271 | 0.36 | -0.0285 | 0.0781 |
| Lung Cancer | TCE | 0.0273 | 0.0223 | 0.22 | -0.0165 | 0.0711 |
| Lung Cancer | VC | 0.0283 | 0.0269 | 0.29 | -0.0246 | 0.0812 |
| Lung Cancer | Benzene | 0.0345 | 0.0302 | 0.25 | -0.0248 | 0.0938 |
| Lung Cancer | TVOC | 0.0231 | 0.0220 | 0.29 | -0.0201 | 0.0663 |
|  |  |  |  |  |  |  |
| Soft Tissue Cancers (N=29) | PCE | -0.0600 | 0.0818 | 0.46 | -0.2207 | 0.1007 |
| Soft Tissue Cancers | TCE | -0.0404 | 0.0668 | 0.55 | -0.1717 | 0.0909 |
| Soft Tissue Cancers | VC | -0.0537 | 0.0806 | 0.51 | -0.2121 | 0.1047 |
| Soft Tissue Cancers | Benzene | -0.0310 | 0.0918 | 0.74 | -0.2114 | 0.1494 |
| Soft Tissue Cancers | TVOC | -0.0398 | 0.0658 | 0.55 | -0.1691 | 0.0895 |
|  |  |  |  |  |  |  |
| Brain Cancer (N=74) | PCE | 0.0169 | 0.0486 | 0.73 | -0.0786 | 0.1124 |
| Brain Cancer | TCE | 0.0087 | 0.0394 | 0.82 | -0.0687 | 0.0861 |
| Brain Cancer | VC | 0.0131 | 0.0477 | 0.78 | -0.0806 | 0.1068 |
| Brain Cancer | Benzene | -0.0144 | 0.0533 | 0.79 | -0.1191 | 0.0903 |
| Brain Cancer | TVOC | -0.0049 | 0.0388 | 0.90 | -0.0811 | 0.0713 |
|  |  |  |  |  |  |  |
| Oral Cancer*** (N=26) | PCE | 0.0688 | 0.0891 | 0.44 | -0.1063 | 0.2439 |
| Oral Cancer | TCE | 0.0409 | 0.0712 | 0.57 | -0.0990 | 0.1808 |
| Oral Cancer | VC | 0.0529 | 0.0866 | 0.54 | -0.1173 | 0.2231 |
| Oral Cancer | Benzene | 0.1605 | 0.0939 | 0.86 | -0.0240 | 0.3450 |
| Oral Cancer | TVOC | 0.0355 | 0.0701 | 0.61 | -0.1022 | 0.1732 |
|  |  |  |  |  |  |  |
| Prostate Cancer (N=18) | PCE | -0.0297 | 0.0957 | 0.76 | -0.2178 | 0.1584 |
| Prostate Cancer | TCE | -0.0522 | 0.0766 | 0.50 | -0.2027 | 0.0983 |
| Prostate Cancer | VC | -0.0508 | 0.0932 | 0.59 | -0.2339 | 0.1323 |
| Prostate Cancer | Benzene | -0.1071 | 0.1025 | 0.30 | -0.3085 | 0.0943 |
| Prostate Cancer | TVOC | -0.0544 | 0.0749 | 0.47 | -0.2016 | 0.0928 |
|  |  |  |  |  |  |  |
| Breast Cancer (females) (N=10) | PCE | 0.0638 | 0.1730 | 0.71 | -0.2761 | 0.4037 |
| Breast Cancer (females) | TCE | 0.0681 | 0.1380 | 0.62 | -0.2031 | 0.3393 |
| Breast Cancer (females) | VC | 0.0739 | 0.1727 | 0.67 | -0.2655 | 0.4133 |
| Breast Cancer (females) | Benzene | 0.1210 | 0.1988 | 0.54 | -0.2696 | 0.5116 |
| Underlying Cause | Log Cum. Exposure | Beta Coefficient | Standard Error | P-value | Lower 95% CL | Upper 95% CL |
| Breast Cancer (females) | TVOC | 0.0595 | 0.1323 | 0.65 | -0.2005 | 0.3195 |
|  |  |  |  |  |  |  |
| Liver Diseases (N=191 | PCE | 0.0168 | 0.0297 | 0.57 | -0.0416 | 0.0752 |
| Liver Diseases | TCE | 0.0175 | 0.0243 | 0.47 | -0.0302 | 0.0652 |
| Liver Diseases | VC | 0.0187 | 0.0293 | 0.52 | -0.0389 | 0.0763 |
| Liver Diseases | Benzene | 0.0171 | 0.0329 | 0.60 | -0.0475 | 0.0817 |
| Liver Diseases | TVOC | 0.0126 | 0.0239 | 0.60 | -0.0344 | 0.0596 |
|  |  |  |  |  |  |  |
| Kidney Diseases (N=37) | PCE | 0.0350 | 0.0682 | 0.61 | -0.0990 | 0.1690 |
| Kidney Diseases | TCE | 0.0283 | 0.0556 | 0.61 | -0.0810 | 0.1376 |
| Kidney Diseases | VC | 0.0329 | 0.0672 | 0.62 | -0.0991 | 0.1649 |
| Kidney Diseases | Benzene | 0.0303 | 0.0754 | 0.69 | -0.1179 | 0.1785 |
| Kidney Diseases | TVOC | 0.0305 | 0.0557 | 0.58 | -0.0790 | 0.1400 |
|  |  |  |  |  |  |  |
| Multiple Sclerosis (N=12) | PCE | -0.0556 | 0.1146 | 0.63 | -0.2808 | 0.1696 |
| Multiple Sclerosis | TCE | -0.0727 | 0.0926 | 0.43 | -0.2547 | 0.1093 |
| Multiple Sclerosis | VC | -0.0777 | 0.1118 | 0.49 | -0.2974 | 0.1420 |
| Multiple Sclerosis | Benzene | -0.1652 | 0.1265 | 0.19 | -0.4138 | 0.0834 |
| Multiple Sclerosis | TVOC | -0.0789 | 0.0908 | 0.39 | -0.2573 | 0.0995 |
|  |  |  |  |  |  |  |
| Amyotrophic Lateral Sclerosis (N=21) | PCE | 0.0836 | 0.0965 | 0.39 | -0.1060 | 0.2732 |
| ALS | TCE | 0.0436 | 0.0773 | 0.57 | -0.1083 | 0.1955 |
| ALS | VC | 0.0724 | 0.0953 | 0.45 | -0.1149 | 0.2597 |
| ALS | Benzene | 0.0579 | 0.1037 | 0.58 | -0.1459 | 0.2617 |
| ALS | TVOC | 0.0702 | 0.0801 | 0.38 | -0.0872 | 0.2276 |
| **Smoking-related Diseases** (not known to be related to solvent exposure) | | | | | | |
| Underlying Cause | Log Cum. Exposure | Beta Coefficient | Standard Error | P-value | Lower 95% CL | Upper 95% CL |
| Stomach Cancer (N=35) | PCE | 0.0474 | 0.0724 | 0.51 | -0.0949 | 0.1897 |
| Stomach Cancer | TCE | 0.0221 | 0.0581 | 0.70 | -0.0921 | 0.1363 |
| Stomach Cancer | VC | 0.0365 | 0.0708 | 0.61 | -0.1026 | 0.1756 |
| Stomach Cancer | Benzene | -0.0087 | 0.0776 | 0.91 | -0.1612 | 0.1438 |
| Stomach Cancer | TVOC | 0.0329 | 0.0588 | 0.58 | -0.0826 | 0.1484 |
|  |  |  |  |  |  |  |
| Cardiovascular Disease† (N=1,391) | PCE | 0.0124 | 0.0111 | 0.27 | -0.0094 | 0.0342 |
| Cardiovascular Disease | TCE | 0.0108 | 0.0091 | 0.23 | -0.0071 | 0.0287 |
| Cardiovascular Disease | VC | 0.0119 | 0.0109 | 0.27 | -0.0095 | 0.0333 |
| Cardiovascular Disease | Benzene | 0.0129 | 0.0123 | 0.30 | -0.0113 | 0.0371 |
| Cardiovascular Disease | TVOC | 0.0111 | 0.0090 | 0.22 | -0.0066 | 0.0288 |
|  |  |  |  |  |  |  |
| Underlying Cause | Log Cum. Exposure | Beta Coefficient | Standard Error | P-value | Lower 95% CL | Upper 95% CL |
| Chronic Obstructive Pulmonary Disease (N=47) | PCE | -0.0611 | 0.0594 | 0.30 | -0.1778 | 0.0556 |
| COPD | TCE | -0.0451 | 0.0485 | 0.35 | -0.1404 | 0.0502 |
| COPD | VC | -0.0585 | 0.0585 | 0.32 | -0.1735 | 0.0565 |
| COPD | Benzene | -0.0683 | 0.0659 | 0.30 | -0.1978 | 0.0612 |
| COPD | TVOC | -0.0456 | 0.0476 | 0.34 | -0.1391 | 0.0479 |

* Biliary passages, liver and gall bladder

** Trachea, bronchus, and lung

*** Buccal cavity and Pharynx.

† Includes diseases of the heart and other diseases of the circulatory system

Notes on the table

Hazard ratios were obtained using the Cox extended model with the log 10 base transformation of cumulative exposure as a time-varying variable and age as the time variable. A small constant (0.001) was added to the cumulative exposure variable to avoid taking the logarithm of zero. The hazard ratios were adjusted by sex, race, rank, and education level. The cumulative exposure variable was lagged 10 years.

The analyses were internal to the Camp Lejeune cohort.

Because of sparse data, regressions were not conducted for the following diseases: male breast cancer, laryngeal cancer, cervical cancer, aplastic anemia, and Parkinson’s disease.
