# Supplementary material for: Evaluation of mortality among marines and navy personnel exposed to contaminated drinking water at USMC base Camp Lejeune: a retrospective cohort study
Source: Environ Health. 2014 Feb 19;13:10. doi: 10.1186/1476-069X-13-10 (PMC3943370; doi:10.1186/1476-069X-13-10)
Supplement: Additional file 4: Figures S1-S3 — Splines of selected causes of death and cumulative exposures. [file 1476-069X-13-10-S4.docx]

**Figure S1**. Spline of kidney cancer and cumulative exposure to all the drinking water contaminants combined (“TVOC”).


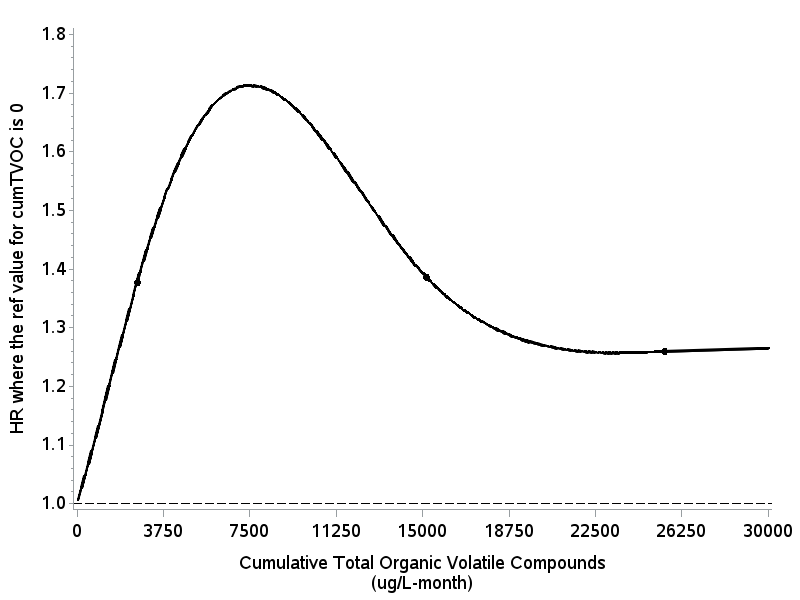


Knots: µg/L-month

5%: 30

25%: 2,612

75% 15,166

95% 25,499

**Figure S2**. Spline of Hodgkin lymphoma and cumulative exposure to trichloroethylene (TCE).


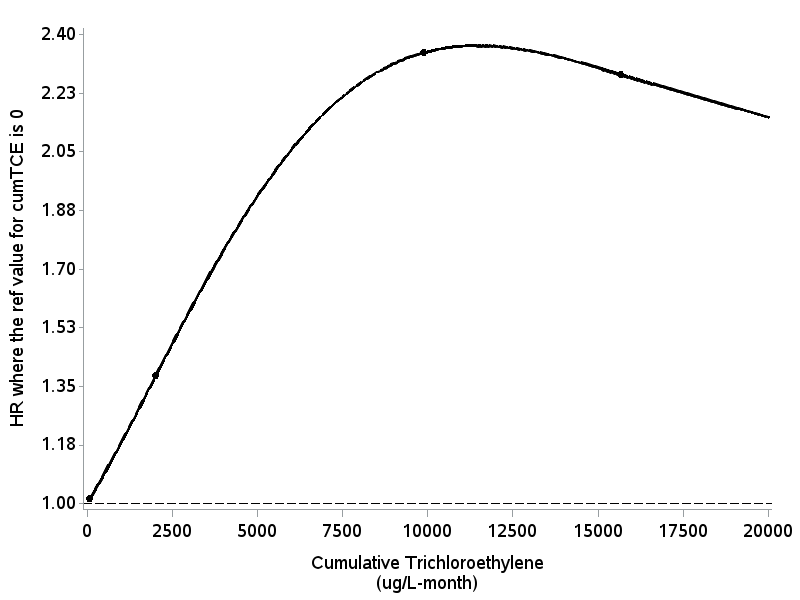


Knots: µg/L-month

5%: 85

25%: 2,017

75% 9.880

95% 15,667

**Figure S3a**. Spline of ALS and cumulative exposure to vinyl chloride.


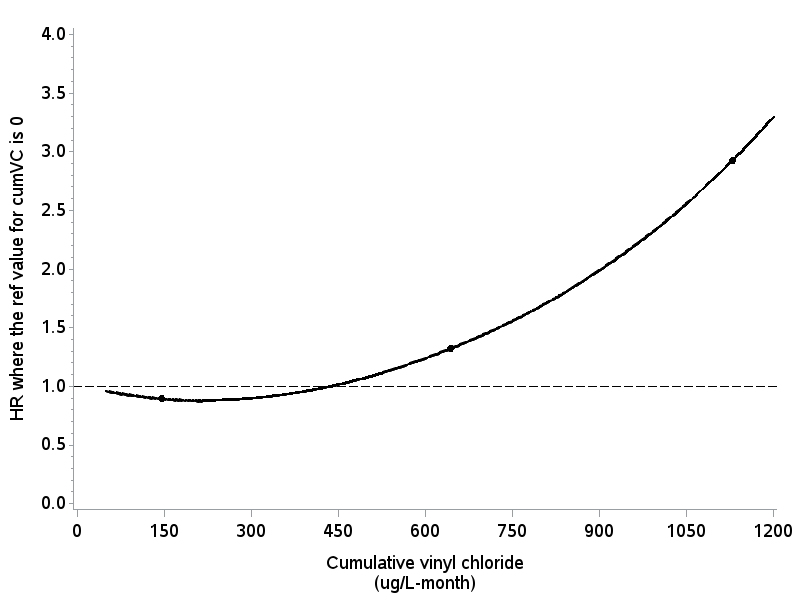


Knots: µg/L-month

5%: 39

25%: 147

75% 645

95% 1,130

**Figure S3b**. Spline of ALS and cumulative exposure to tetrachloroethylene (PCE).


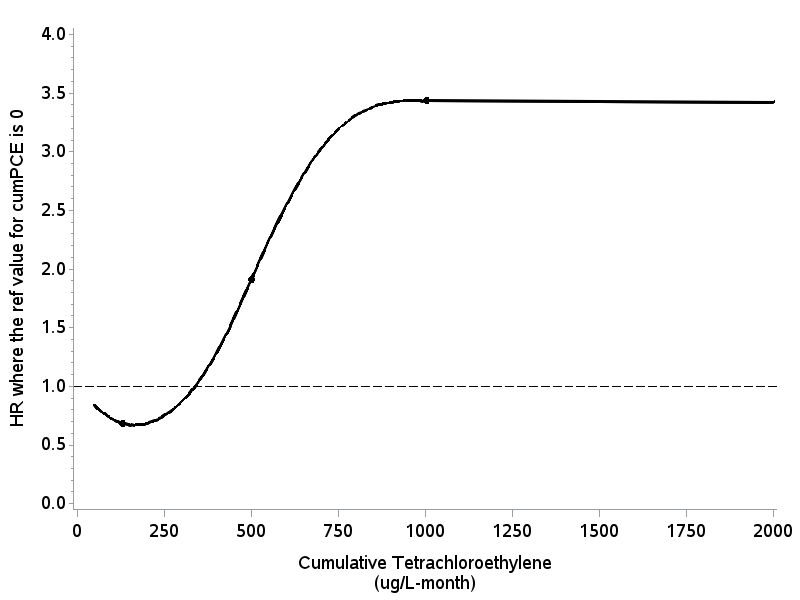


Knots: µg/L-month

5%: 21

25%: 130

75% 501

95% 1,005
